# Biofilm-targeted liposomal curcumin delivery system for anti-caries therapy

**DOI:** 10.3389/fcimb.2026.1808450

**Published:** 2026-04-29

**Authors:** Yao Chen, Jing Li, Yongjian Sun, Xin Yue, Xiao-Han Tian, Feng Liu, Da-Yuan Wang, Jing Shen

**Affiliations:** 1 Department of Operative Dentistry and Endodontics, Tianjin Stomatological Hospital, School of Medicine, Nankai University, Tianjin, China; 2 Tianjin Key Laboratory of Oral and Maxillofacial Function Reconstruction & Stomatology Institute of Nankai University, Tianjin, China; 3 Department of International VIP Dental Clinic, Tianjin Stomatological Hospital, School of Medicine, Nankai University, Tianjin, China; 4 State Key Laboratory of Medicinal Chemical Biology, Key Laboratory of Functional Polymer Materials, Ministry of Education, Institute of Polymer Chemistry, College of Chemistry, Nankai University, Tianjin, China; 5 Key Laboratory of Industrial Fermentation Microbiology, Ministry of Education, College of Biotechnology, Tianjin University of Science and Technology, Tianjin, China

**Keywords:** biofilm-targeted, caries, curcumin, eliminating biofilms, liposomes

## Abstract

**Introduction:**

Dental caries, driven by acidogenic biofilms, remains a major global health challenge. Current chemical treatments, such as chlorhexidine and fluoride, can disrupt oral microbial homeostasis and cause adverse effects, including tooth discoloration, dentin hypersensitivity, and taste disturbances. Curcumin, a natural photosensitizer, exhibits antibacterial activity and favorable biocompatibility, however, its clinical application is limited by poor stability, low aqueous solubility, and restricted biofilm penetration. There is an urgent need to develop innovative therapeutic strategies to enhance curcumin transport into acidic cariogenic biofilms.

**Methods:**

We developed a pH-responsive liposomal delivery system (Cur/DCPA-H_2_O) engineered to penetrate acidic cariogenic biofilms and enhance curcumin transport. The physicochemical characterization of Cur/DCPA-H_2_O was performed using a UV-1800 spectrophotometer, transmission electron microscopy (TEM), and dynamic light scattering (DLS). Biocompatibility was assessed by Cell Counting Kit-8 (CCK-8) assays, hemolysis tests, and Live/Dead cell staining. The antibacterial efficacy *in vitro* and *ex vivo* was evaluated using colony-forming unit (CFU) counting, scanning electron microscopy (SEM), confocal laser scanning microscopy (CLSM), and crystal violet (CV) staining. An *in vivo* caries model was established to assess the therapeutic efficacy of Cur/DCPA-H_2_O, using micro-computed tomography (micro-CT), Keyes' scoring, and 16S rRNA sequencing.

**Results:**

The liposomes exploit charge reversal to interact with representative caries-associated bacteria (Streptococcus mutans and the early colonizer Streptococcus sanguinis), enabling deep biofilm penetration. Upon light irradiation, Cur/DCPA-H_2_O was observed to generate reactive oxygen species (ROS), which may contribute to partial disruption of the biofilm matrix and reduced bacterial viability *in vitro*. In a rat caries model, treatment with Cur/DCPA-H_2_O under light irradiation reduced caries severity and decreased lesion depth by approximately 50%. It also shifted the oral microbiome composition toward a less dysbiotic profile, as confirmed by 16S rRNA sequencing.

**Discussion:**

This study demonstrates that a biofilm-targeted, pH responsive liposomal curcumin delivery system may provide a safe and effective strategy for caries prevention, highlighting the potential of natural therapeutics to modulate pathogenic biofilms with limited impact on the overall microbial community.

## 1 Introduction

Caries is one of the most prevalent chronic non-communicable diseases worldwide, affecting nearly 3 billion individuals and resulting in an estimated US$23.55 billion in indirect costs (Ealla et al., 2024). The World Health Organization lists dental caries as one of the three major diseases to be prevented and controlled, alongside cancer and cardiovascular diseases (Peres et al., 2019). From an etiological perspective, caries is a biofilm-mediated pathological process characterized by progressive demineralization of dental hard tissues (Yeh et al., 2025). Cariogenic bacteria, particularly Streptococcus mutans, play a central role by metabolizing dietary carbohydrates into organic acids, leading to localized acidification within dental biofilms. Additionally, Streptococcus sanguinis has been reported to cooperate with *S. mutans* within biofilms and may influence microbial interactions that contribute to caries progression (Liu et al., 2021). The accumulation of these organic acids results in a localized acidic microenvironment within biofilms. The acidic microenvironment (pH < 5.0) is a characteristic feature of carious lesions (Deus and Ouanounou, 2022), distinct from the mild pH fluctuations (pH 6.2-7.6) observed in healthy oral tissues (He et al., 2023). Such local acidification can disrupt the balance of calcium and phosphate ions, promote enamel demineralization and may ultimately result in irreversible tooth cavities or dentin hypersensitivity. Therefore, strategies for caries combating often focus on controlling pathogenic biofilms.

Currently, clinical treatment mainly relies on fluoride and antimicrobial agents such as chlorhexidine. While these approaches are effective in reducing bacterial load, their long-term use may be associated with adverse effects. Excessive use of fluoride can cause tooth discoloration, pulp irritation and potential cytotoxicity (Li et al., 2025b). Long-term use of chlorhexidine can lead to oral mucosal damage, taste alteration and increased antimicrobial resistance (Liu et al., 2021). Emerging alternatives, including antimicrobial peptides, metal-based agents, and natural compounds, have been explored to overcome these limitations. Unfortunately, these approaches may still face challenges related to cost, stability, potential cytotoxicity, or limited specificity. Metal ion antibacterial agents may discolor teeth and induce oxidative stress (Yin et al., 2020); antimicrobial peptides are costly, unstable and prone to cause dysbiosis (Ying et al., 2024). Therefore, a safe and effective method for preventing dental caries is needed, which can penetrate biofilm, kill bacteria, and degrade the extracellular polysaccharides (EPS) matrix while inhibiting demineralization.

Recently, natural antibacterial agents have attracted increasing attention owing to their low cost, minimal toxicity, and favorable biocompatibility (Li et al., 2025a). Among them, curcumin—a natural polyphenolic compound extracted from *Curcuma longa*—has emerged as a promising candidate for caries prevention and treatment due to its potent antibacterial properties and excellent safety profile (Kashi et al., 2025). On the one hand, curcumin can modulate the metabolic pathways of *S. mutans* and *S. sanguinis* (Guzman-Flores et al., 2024), disrupt the structure of EPS (Kashi et al., 2024), and inhibit bacterial proliferation in acidic environments (Sekiya et al., 2019). On the other hand, as a natural photosensitizer, curcumin can generate reactive oxygen species (ROS) under light irradiation, leading to oxidative damage of bacterial membranes and nucleic acids, ultimately resulting in bacterial death (Pramana et al., 2024). However, the clinical application of curcumin remains limited by its poor aqueous solubility, low bioavailability, and chemical instability (Ashwini et al., 2025). These limitations significantly reduce its therapeutic efficacy in complex oral environments.

Nanotechnology-based delivery systems offer promising strategies to enhance the delivery efficiency and therapeutic performance of natural antimicrobial agents (Cheng et al., 2025). Among them, biofilm-targeting liposomal systems are particularly attractive due to their high biocompatibility, favor drug stability, and ability to adapt to the dynamic conditions of the oral microenvironment (Wang et al., 2021). In our previous work, we developed biocompatible self-targeting synthetic liposomes composed of DCPA-H_2_O, in which complexed water provides pH-responsive functionality. These liposomes possess a slightly negative surface charge under physiological conditions to maintain stability. However, when the environmental pH drops below 7.0, protonation of the complexed water in DCPA-H_2_O induces a shift toward a positive charge, which enhances electrostatic interactions with negatively charged bacterial surfaces and biofilms. DCPA-H_2_O liposomes accumulate at acidic biofilm sites, where they interact with and penetrate the biofilm structure, followed by disassembly and cargo release (Wang et al., 2021, Wang et al., 2022b).

**Scheme 1:**
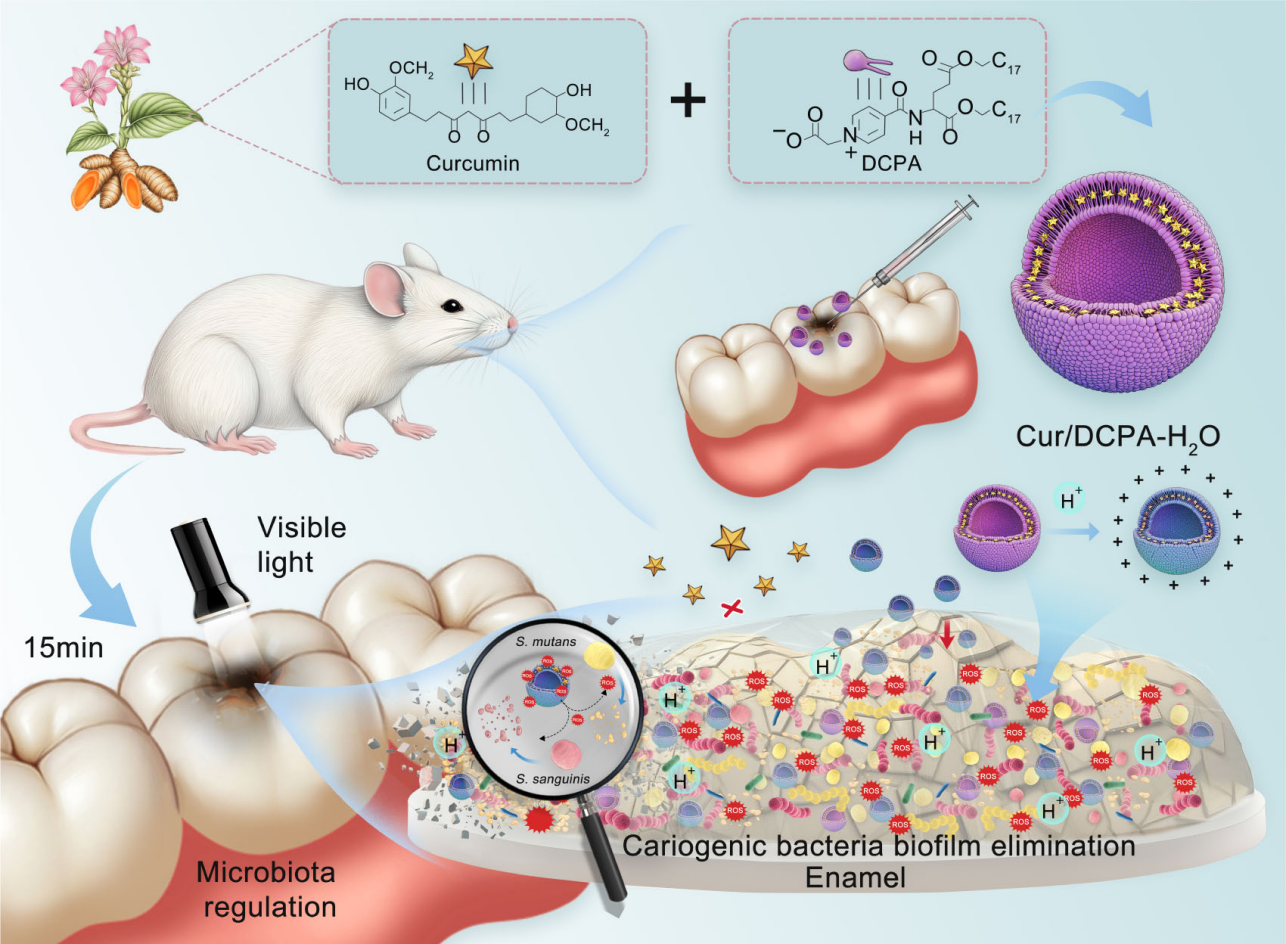
Schematic illustration of curcumin-loaded liposomes (Cur/DCPA-H_2_O) mediating antibacterial photodynamic therapy for caries treatment. In acidic biofilm environments, the liposomes undergo rapid protonation, acquiring a positive charge that enables targeted delivery of curcumin to negatively charged biofilms. Upon light irradiation, ROS are generated, disrupting the bacterial extracellular matrix and inhibiting biofilm growth.

In this context, we developed curcumin-loaded DCPA-H_2_O liposomes (Cur/DCPA-H_2_O) to enhance the delivery of curcumin into plaque biofilms. The system is designed to respond to acidic microenvironments generated by cariogenic biofilms, which may promote interaction with bacterial surfaces through electrostatic effects (see **Scheme 1**). Upon light irradiation, curcumin generates abundant ROS, effectively eliminating surrounding bacterial biofilm (also see **Scheme 1**), inhibiting the development of dental caries and promoting enamel repair. To verify these hypotheses, we evaluated the antimicrobial activity of Cur/DCPA-H_2_O against *S. mutans* and *S. sanguinis in vitro*, as well as its therapeutic effects *in ex vivo* human plaque samples and *in vivo* caries animal models. The results demonstrated that Cur/DCPA-H_2_O can target and eradicate plaque bacterial biofilm, effectively inhibiting and reversing caries progression *in vivo*. Notably, both *in vitro* and *in vivo* assessments confirmed the excellent biocompatibility of this system. Overall, this strategy provides a promising new approach for combating dental caries.

## 2 Materials and methods

### 2.1 Materials

Methanol (MeOH), dimethyl sulfoxide (DMSO), tetrahydrofuran (THF) were purchased from Tianjin Bohua Chemical Reagent Co. Ltd (Tianjin, China). Boc-L-glutamic acid, 1-octadecanol, isonicotinic acid, bromoacetic acid, trifluoroacetic acid (TFA) and curcumin were purchased from Heowns Biochem Technologies LLC (Tianjin, China). Triton X-100 was purchased from Beijing Labgic Technology Co., Ltd (Beijing, China). Nile red was purchased from Samgon Biochemical Technology Co., Ltd (Shanghai, China). Streptococcus mutans (UA159), Streptococcus sanguinis (BNCC354356), the mouse fibroblast cell line L929 and MC3T3-E1 Subclone 14 cells were provided by the Tianjin Key Laboratory of Oral and Maxillofacial Function Reconstruction in Tianjin Stomatological Hospital (Tianjin, China). Brain heart infusion (BHI) medium and BHI agar plates were purchased from Haibo Biotechnology Co., Ltd (Qingdao, China). Phosphate buffer solution (PBS), α-MEM medium, fetal bovine serum (FBS), penicillin-streptomycin (PS) and trypsin were obtained from Gibco (Grand Island, USA). Calcein-AM/PI assay kit, CCK-8 reagent, immunostaining fixation buffer, immunostaining wash buffer, Actin-Tracker Green-488, immunofluorescence secondary antibody dilution buffer and DAPI were purchased from Beyotime Biotechnology Co., Ltd (Shanghai, China).

### 2.2 Preparation of Cur/DCPA-H_2_O

The lipid DCPA and curcumin were incorporated into the shell. DCPA (10 mg), and curcumin (1 mg) were dissolved in 1 mL of THF. The solution was subsequently rapidly injected into 10 mL of deionized water under stirring at 600 rpm and maintained at 50 °C. After stirring for approximately 10 min, the solution was transferred to a dialysis bag (MWCO 50 kDa) and dialyzed against deionized water for 24 h to remove the organic solvents and molecules that did not participate in the self-assembly. The liposome suspension was filtered through a 0.45 μm syringe filter membrane (Merck, USA) to remove liposomes larger than 0.45 μm. Finally, the liposome suspension was concentrated by low-speed ultrafiltration using an ultrafiltration centrifuge tube (15 mL, MWCO 10 kDa, polytetrafluoroethylene membrane) for 10 min at 1500 rpm. To determine the concentration of the liposome, 500 μL of the liposome suspension was freeze-dried and weighed. The drug loading content (DLC) of Cur/DCPA-H_2_O liposomes was calculated using the formula:


$$DLC\left(\%\right)=\frac{\text{Weight of loaded curcumin }}{\text{Total weight of lipid and loaded curcumin}}\times 100\%$$

Before the experiment, the liposome suspension was sterilized by filtration (MILLEX^®^GP 0.22 μm).

### 2.3 Characterization of Cur/DCPA-H_2_O liposomes

The hydrodynamic diameter, polydispersity index (PDI), and zeta potential of the liposomes (0.05 mg·mL^-1^) at different pH values were measured using dynamic light scattering (Malvern Zetasizer Nano-ZS90) at 25 °C. The morphology of Cur/DCPA-H_2_O liposomes was observed by transmission electron microscopy (TEM, Talos™ F200C) at 200 kV after staining. Three independent measurements were performed using separately prepared liposome suspensions for each replicate. UV-Vis absorption spectra of DCPA-H_2_O and Cur/DCPA-H_2_O were recorded using a UV-1800 spectrometer (Shimadzu, Shanghai, China). Curcumin solutions at different concentrations in DMSO were also analyzed with the UV-1800 spectrometer to construct calibration curves for quantitative determination.

### 2.4 Determination of ROS generation by Cur/DCPA-H_2_O liposomes

9,10-Anthracenediyl-bis(methylene) dimalonic acid (ABDA, MCE) was used as a ROS probe. To evaluate the ROS-generating capability, Cur/DCPA-H_2_O liposomes (12.5 mg·mL^-1^) and curcumin dissolved in DMSO (12.5 mg·mL^-1^) were separately mixed with ABDA. The mixtures were irradiated with visible light (28 mW·cm^-2^) at 1-minute intervals, and the UV-Vis absorption spectra were recorded at 400 nm to monitor ROS generation.

### 2.5 Determination of pH in bacterial suspensions over time

Two milliliters of bacterial suspension (1 × 10^8^ CFU·mL^-1^, n = 3) of *S. mutans* or *S. sanguinis* were added to 20 mL of BHI medium (supplemented with 1% sucrose). At 0, 2, 4, 6, 8, 10, 12 and 24 h, 2 mL aliquots were removed for pH measurement.

### 2.6 *In vitro* evaluation of the antibacterial effect of Cur/DCPA-H_2_O liposomes

The antibacterial activity of Cur/DCPA-H_2_O liposomes was evaluated against *S. mutans* and *S. sanguinis* in both planktonic cultures and biofilm states. Bacterial suspensions and biofilms were treated under various conditions, including PBS, free curcumin, and Cur/DCPA-H_2_O, with or without visible light irradiation (28 mW·cm^-2^). The effects on bacterial viability, biofilm structure, and biomass were assessed using colony counting, confocal laser scanning microscope (CLSM) imaging, scanning electron microscopy (SEM), and crystal violet (CV) staining.

#### 2.6.1 Bacterial culture and biofilm preparation

*S. mutans* (UA159) and *S. sanguinis* (BNCC354356) were resuscitated in BHI medium (supplemented with 1% sucrose) and cultured under microaerobic conditions with 5% CO_2_ at 37 °C. Bacterial cultures in the logarithmic growth phase were used for all experiments. Suspensions were diluted in PBS to the required concentrations, which were determined using a Bürker-Türk counting chamber. For biofilm preparation, 1 mL bacteria in suspension (1 × 10^8^ CFU·mL^-1^) in PBS was added to confocal culture dishes and incubated at 37 °C for 1 h to allow adherence. Subsequently, non-adherent bacteria were removed by washing twice with 1 mL PBS, and 1 mL of BHI medium (supplemented with 1% sucrose) was added to each dish. Biofilms were incubated for 48 h at 37 °C.

#### 2.6.2 Targeting of Cur/DCPA-H_2_O to planktonic bacteria

*S. mutans* and *S. sanguinis* suspensions (1 × 10^6^ CFU·mL^-1^, n = 3) were cocultured with PBS (negative control) or Nile red-loaded Cur/DCPA-H_2_O liposomes. After 2 h incubation at 37 °C, wells were washed twice with PBS. Resuspended bacterial suspensions were mixed with 1.5 μL SYTO 9 (FUSHENBIO) in the dark for 30 min. A drop of each suspension was placed on a glass slide and examined under a CLSM (Leica TCS SP8, Wetzlar, Germany).

#### 2.6.3 *In vitro* antimicrobial test of planktonic bacteria

Bacterial suspensions (1 × 10^6^ CFU·mL^-1^, n = 3) were incubated with Cur/DCPA-H_2_O at different concentrations (0, 20, 40, and 80 μg·mL^-1^) for 1 h, followed by white light irradiation (28 mW·cm^-2^) for 15 min. The suspensions were serially diluted, plated on BHI agar and blood agar plates. After incubation at 37 °C for 24 h, colonies were counted to assess antibacterial efficacy.

#### 2.6.4 Scanning electron microscopy

Suspensions of *S. mutans* and *S. sanguinis* (10^4^CFU·mL^-1^, n = 3) were cultured on silicon wafers (5 × 5 mm) under different treatment conditions for 2 h: PBS light (+), Cur light (+), Cur/DCPA-H_2_O light (-), and Cur/DCPA-H_2_O light (+). Samples requiring light exposure were irradiated with white light (28 mW·cm^-2^) for 15 min. For biofilm studies, 48-hour *S. mutans* and *S. sanguinis* biofilms were cultured on silicon wafers under the same treatment conditions for 12 h, followed by white light irradiation (28 mW·cm^-2^) for 15 min where applicable. All samples were fixed in 5% glutaraldehyde for 2 h, then dehydrated in 10%, 30%, 50%, 70%, 90%, and 100% methanol for 15 min at each step. After critical point drying with CO_2_, the samples were gold-coated and observed using a TM4000PLUS scanning electron microscope (Hitachi, Japan) to visualize the detailed surface structures.

#### 2.6.5 Biofilm Penetration of Cur/DCPA-H_2_O

Forty-eight-hour *S. mutans* and *S. sanguinis* biofilms were rinsed twice with PBS and then exposed to PBS or Nile red-loaded Cur/DCPA-H_2_O for 2 h. Following exposure, the biofilms were washed twice with PBS. 1 mL of the resuspended bacterial suspension was mixed with 1.5 μL SYTO 9 (FUSHENBIO) and incubated in the dark for 15 min. After two additional PBS washes, the biofilms were imaged using CLSM (Leica TCS SP8, Wetzlar, Germany). In a separate experiment, 48-hour *S. mutans* and *S. sanguinis* biofilms were rinsed twice with PBS and exposed to PBS or Cur/DCPA-H_2_O for 12 h, followed by white light irradiation (28 mW·cm^-2^) for 15 min. After treatment, the biofilms were washed twice with PBS. 1 mL of the resuspended bacterial suspension was then mixed with 3 μL of dye mixture (SYTO 9: PI = 1:3) and incubated in the dark for 15 min. Following two additional PBS washes, the biofilms were imaged using CLSM (Leica TCS SP8, Wetzlar, Germany).

#### 2.6.6 Crystal violet staining

Forty-eight-hour *S. mutans* and *S. sanguinis* biofilms were cultured in 24-well plates under different treatment conditions for 12 h, followed by white light irradiation (28 mW·cm^-2^) for 15 min (n = 3). After treatment, the biofilms were fixed with absolute methanol for 30 min and then stained with 0.1% crystal violet solution for 30 min. The biofilms were washed three times with water, and the bound dye was extracted with 30% acetic acid for 10 min. The extracted solution was transferred to a 96-well plate, and absorbance was measured at 590 nm using a microplate reader.

### 2.7 *Ex vivo* killing oral biofilm collected from dental caries patients

Dental caries plaque samples were collected from carious lesions of patients at the Department of Operative Dentistry and Endodontics, Tianjin Stomatological Hospital, with approval from the Ethics Committee of Tianjin Stomatological Hospital (PH-2023-J-025). The inclusion criteria were affected teeth presenting with sub-enamel destruction or frank cavitation on pit-and-fissure or smooth surfaces, with softness of the cavity base or walls verified by a dental CPI probe. The exclusion criteria were teeth with pulp infection, antibiotics or other oral antimicrobial agents in the previous 3 months, and currently undergoing dental treatment (Dong et al., 2025). An endodontist collected 5 enamel caries samples. We used sterile dental excavators to obtain samples from enamel. Plaque samples were suspended in 1 mL sterile PBS and stored at -80 °C until used.

#### 2.7.1 Evaluation of antibacterial effectiveness

The plaque samples were randomly allocated across treatment groups: PBS light (+), Cur light (+), Cur/DCPA-H_2_O light (-), and Cur/DCPA-H_2_O light (+). Colony counting, CV staining, and SEM imaging were performed to evaluate overall antibacterial effect. Species-level microbial composition was further analyzed using 16S rRNA sequencing.

#### 2.7.2 Microbiome analysis

For sequencing analysis, five samples per group were resuscitated and cultured in BHI medium (supplemented with 1% sucrose). The samples were then exposed to treatment conditions corresponding to those used in the *in vitro* antibacterial experiments and subsequently processed for 16S rRNA sequencing. After DNA extraction, PCR amplification, and product purification, the V3-V4 region was amplified using primers 338F (5′-ACTCCTACGGGA GGCAGCAG-3′) and 806R (5′-GGACTACHVGGGTWTCTAAT-3′). Libraries were sequenced on the Illumina NextSeq platform, and data analysis was performed using Majorbio Cloud (www.majorbio.com).

### 2.8 *In vitro* assessment of Cur/DCPA-H_2_O biocompatibility

#### 2.8.1 Hemolysis test of red blood cells

Blood was collected from healthy mice via retro-orbital bleeding and centrifuged at 1500 × g for 15 min to separate serum. The erythrocytes were washed three times with PBS and resuspended in PBS to prepare a 4% (v/v) erythrocyte suspension. The positive control group was treated with 0.5% Triton X-100, while the negative control group received PBS. For the experimental groups, equal volumes of the erythrocyte suspension were mixed with different concentrations of Cur/DCPA-H_2_O and incubated at 37 °C for 2 h. The mixtures were then centrifuged at 1500 × g for 15 min, and 100 μL of each supernatant was transferred to a 96-well plate. The absorbance at 540 nm was measured using a Tecan Spark microplate reader (Mannnedorf, Switzerland). The hemolysis rate was calculated as follows:


$$Hemolysis\left(\%\right)=\frac{OD_{sample}-OD_{negative\;control}}{OD_{positive\;control}-OD_{negative\;control}}\times 100\%$$

All biocompatibility assays were performed in triplicate.

#### 2.8.2 Cell culture

The cytotoxic effects of Cur/DCPA-H_2_O were evaluated through experiments conducted on the L929 cell line and MC3T3-E1 cells. Both L929 and MC3T3-E1 cells were cultured in α-MEM medium supplemented with 10% fetal bovine serum and 1% penicillin-streptomycin solution. The cells were incubated at 37°C in a humidified 5% CO_2_ atmosphere, with the medium refreshed every other day. Cell growth density was monitored via an inverted microscope, and subculturing was performed once cell confluency reached 80%. All biocompatibility assays were performed in triplicate.

#### 2.8.3 Live/dead cell assay

Cell viability was further evaluated using the calcein-AM/PI assay kit. L929 cells were incubated with the calcein/PI buffer for 30 min, and live (green) and dead (red) cells were observed under a Ti-S inverted fluorescence microscope (Nikon, Japan). Quantitative fluorescence analysis was normalized to strain-specific PBS controls to account for inherent differences in biofilm architecture between the two species. All biocompatibility assays were performed in triplicate.

#### 2.8.4 Cell viability of Cur/DCPA-H_2_O assay

L929 cells were seeded into 96-well plates at 5 × 10^4^cells per well in 100 μL medium. When cells reached 80% confluence, different concentrations of Cur/DCPA-H_2_O (0, 10, 20, 40, 80, and 160 μg·mL^-1^ curcumin) were added and incubated for 12 h. Cells were then exposed to visible light (28 mW·cm^-2^) for 15 min and further cultured for 12 h. After incubation, 10% CCK-8 reagent was added for 2 h in the dark, and absorbance at 450 nm was measured using a microplate reader. Cell survival rate was calculated as follows:


$$Cell\;viability\left(\%\right)=\frac{OD_{sample}-OD_{negative\;control}}{OD_{positive\;control}-OD_{negative\;control}}\times 100\%$$

All biocompatibility assays were performed in triplicate.

#### 2.8.5 Cytoskeleton staining of MC3T3-E1 cells

MC3T3-E1 cells were co-incubated with Cur/DCPA-H_2_O for 24 h, then washed twice with PBS. Cells were fixed with immunostaining fixation buffer for 15 min at room temperature, followed by two washes with immunostaining wash buffer. Cells were incubated with Actin-Tracker Green-488 diluted in immunofluorescence secondary antibody dilution buffer for 30 min at room temperature in the dark. After two additional washes, cells were stained with DAPI for 5 min at room temperature in the dark, washed twice with PBS, and imaged using a Ti-S inverted fluorescence microscope (Nikon, Japan). All biocompatibility assays were performed in triplicate.

### 2.9 Evaluation of the therapeutic efficacy of Cur/DCPA-H_2_O on dental caries *in vivo*

#### 2.9.1 Animals

Sprague–Dawley (SD) male rats (28 days old) were purchased from SPF Biotechnology Co., Ltd (Beijing, China). All animals were housed in the on-site animal facility at Zhongxin Huake Biotechnology Co., Ltd (Tianjin, China). Animal experiments were conducted in accordance with the Guidelines for Care and Use of Laboratory Animals, and approved by the Ethics Committee of the Tianjin Stomatological Hospital (PA2023-B-018).

#### 2.9.2 Establishment of rat dental caries model

Twenty male SD rats (28 days old) were initially fed antibiotic-supplemented feed and water for three consecutive days (days 28 to 31). Clearance of endogenous oral bacteria was confirmed via oral swab sampling. For the next three consecutive days (days 31 to 34), exogenous *S. mutans* was inoculated twice daily. Throughout the study, rats were fed a Keyes 2000 high-sugar diet and 5% sucrose solution ad libitum.

#### 2.9.3 Treatment of dental caries in rats

Once infection was established, rats were randomly divided into four groups (5 rats per group): PBS light (+), Cur light (+), Cur/DCPA-H_2_O light (-), and Cur/DCPA-H_2_O light (+). Topical treatments (100 μL) were applied orally once daily starting on day 34, with 1-minute coating per tooth surface, followed by 15 minutes of lateral white light irradiation (28 mW·cm^-2^). Treatments were administered for 4 weeks by the same experimenter. At the end of the experimental period, oral microbiota samples were collected with sterile swabs. Rats were euthanized using excessive CO_2_. The upper and lower jaws were dissected, and tissues from the lips, tongue, small intestine, stomach, heart, liver, spleen, lungs, and kidneys were collected and fixed in 4% paraformaldehyde for 24 h.

#### 2.9.4 Evaluation of therapeutic efficacy

Micro-CT scans were performed on maxillary molars to assess carious lesions. H&E staining of collected tissues evaluated *in vivo* biocompatibility. Rat maxillary and mandibular bone specimens were stained with 0.4% ammonium purple urea solution for 12 h under light protection, rinsed, and dried at room temperature for 24 h. Mandible specimens were halved along the maxillary bone and near-distal sagittal planes of the molars using a high-speed plaster model cutter. Caries lesions were classified as:

Initial lesions: affecting enamel only, ≤ 1/4 of dentin (blue arrows).

Moderate lesions: affecting 1/4-3/4 of dentin (yellow arrows).

Extensive lesions: affecting >3/4 of dentin (red arrows).

The Larson-modified Keyes scoring system was applied: 1-enamel only; 2-slight dentinal (<1/4 dentin); 3-moderate dentinal (1/4-3/4 dentin); 4-extensive dentinal (>3/4 dentin). Red-stained areas indicated carious tissue, while lightly or non-stained regions represented healthy dentin.

#### 2.9.5 Impact of Cur/DCPA-H_2_O liposomes on oral microbiome homeostasis

Oral microbiota samples were collected, frozen on dry ice, and sent for 16S rRNA sequencing. After DNA extraction, PCR amplification, and product purification, the V3-V4 region was amplified using primers 338F (5′-ACTCCTACGGGAGGCAGCAG-3′) and 806R (5′-GGACTACHVGGGTWTC TAAT-3′). Libraries were sequenced on the Illumina NextSeq platform, and data analysis was performed using Majorbio Cloud (www.majorbio.com).

### 2.10 Statistical analysis

All assays were independently repeated three times. Results are expressed as mean ± SD. The variance homogeneity of all data was verified by the Brown-Forsythe test. For comparisons between two groups, independent samples t-tests were performed. For comparisons among multiple groups, one-way analysis of variance (ANOVA) followed by Tukey’s *post hoc* test was applied. Differences were considered statistically significant at p < 0.05. *p < 0.05; **p < 0.01; ***p < 0.001; ****p < 0.0001. For microbiome data, alpha diversity indices including observed Ace and Shannon index were calculated with Mothur v1.30.1 based on the species information. The similarity among the microbial communities in different samples was determined by principal coordinate analysis (PCoA) based on Bray-curtis dissimilarity using Vegan v2.5–3 package. The ANOSIM analysis was used to assess the percentage of variation explained by the treatment along with its statistical significance using Vegan v2.5–3 package. The linear discriminant analysis (LDA) effect size (LEfSe) was performed to identify the significantly abundant taxa (phylum to genera) of bacteria among the different groups (LDA score > 2, P < 0.05). All analyses were implemented on the Majorbio Cloud Platform (www.majorbio.com).

## 3 Results and discussion

### 3.1 Synthesis of biofilm-targeting lipid DCPA

DCPA was synthesized according to the previously reported method, and the chemical structure was confirmed by nuclear magnetic resonance spectroscopy (Wang et al., 2021) (see **Supplementary Figure S1**). In brief, Boc-L-glutamic acid was reacted with 1-octadecanol to introduce hydrophobic tail groups. Subsequently, 50% trifluoroacetic acid (TFA) was used to remove the Boc-protection at 0 °C, thereby exposing the terminal amino group, which was then coupled with the carboxyl group of isonicotinic acid. Finally, the nitrogen atom in the pyridine ring was quaternized with bromoacetic acid under elevated temperature conditions to yield DCPA (see **Supplementary Figure S1**).

### 3.2 Preparation and characterization of the biofilm-targeted curcumin-loaded liposomes, Cur/DCPA-H_2_O

**Figure 1:**
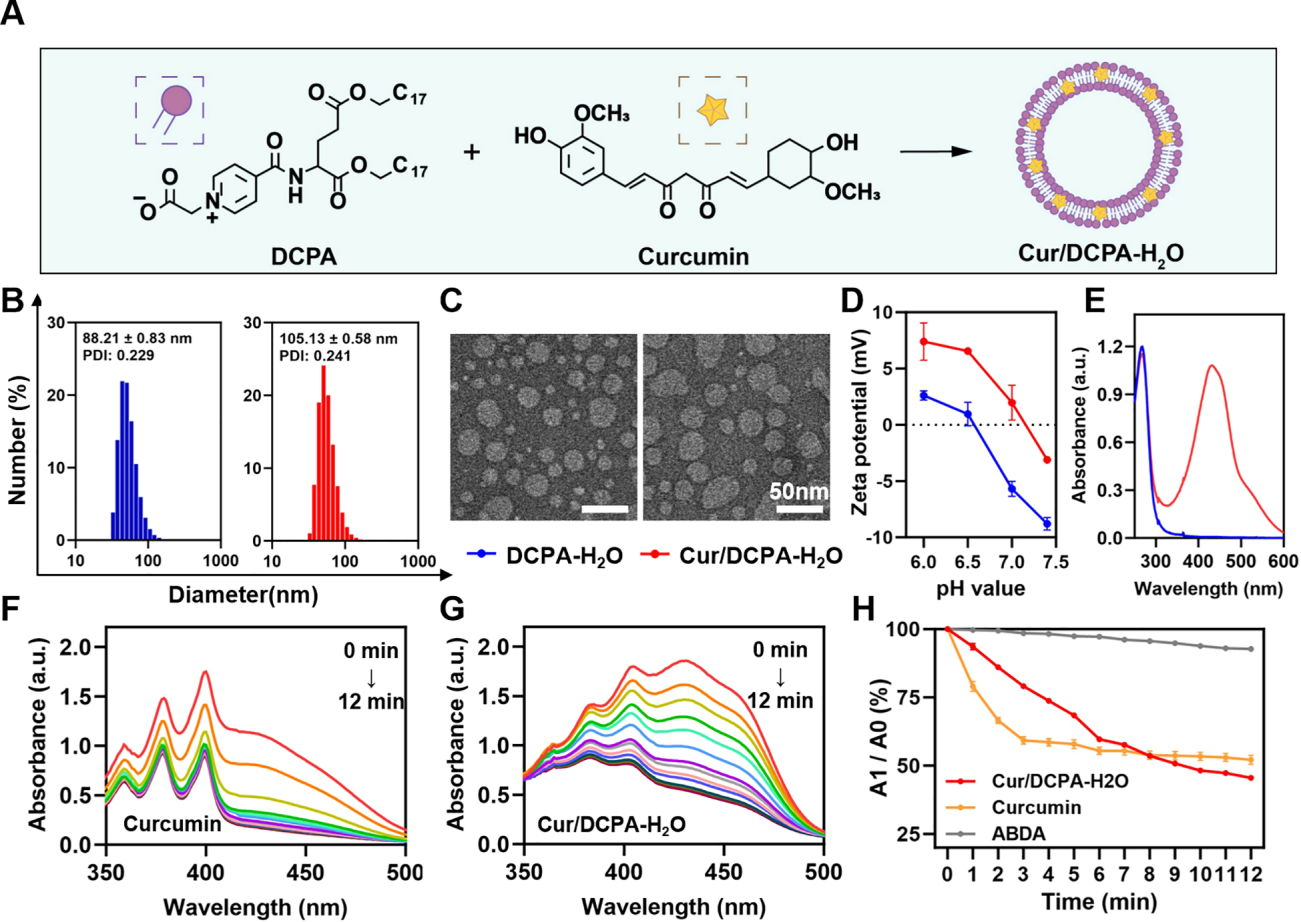
Preparation and characterization of Cur/DCPA-H_2_O. **(A)** Schematic illustration of preparation curcumin loaded DCPA-H_2_O liposomes, Cur/DCPA-H_2_O. **(B)** Diameter distributions of DCPA-H_2_O and Cur/DCPA-H_2_O liposomes (0.1 mg mL^-1^, respectively) measured in water by dynamic light scattering. **(C)** As same as panel B, now for TEM micrographs of DCPA-H_2_O and Cur/DCPA-H_2_O liposomes. **(D)** Zeta potentials of DCPA-H_2_O and Cur/DCPA-H_2_O liposomes (0.1 mg mL^-1^, respectively) suspended in a phosphate buffer (pH 7.4, 5 mM NaH_2_PO_4_ and 5 mM Na_2_HPO4). Zeta potentials were measured 2 min after suspending. **(E)** UV-vis spectra between 260 nm and 600 nm of curcumin, DCPA-H_2_O and Cur/DCPA-H_2_O. **(F)** UV-vis spectra between 350 nm and 500 nm of curcumin coculture with ABDA as a function of visible light (28 mW cm^-2^) irradiation. **(G)** As same as panel **(F)**, now for Cur/DCPA-H_2_O liposomes coculture with ABDA. **(H)** Cumulative ROS generated as a function of visible light irradiation time from curcumin and Cur/DCPA-H_2_O liposomes, respectively. Generated ROS were calculated from panel **(F)** and **(G)**. Error bars denote the standard deviations over triplicate measurements with separately prepared liposomes.

Curcumin-loaded nanoliposomes were fabricated using an injection method as previously described (Wang et al., 2021). Briefly, DCPA and curcumin were dissolved at various weight ratios in 1 mL of THF and injected into 10 mL of deionized water to form curcumin-loaded DCPA-H_2_O liposomes, Cur/DCPA-H_2_O (**Figure 1A**). Notably, when the curcumin-to-DCPA ratio exceeded 0.1, both the average hydrodynamic diameter and polydispersity index (PDI) increased substantially (**Supplementary Table S1**), reaching levels unsuitable for biofilm penetration and reticuloendothelial evasion (Gao et al., 2020). In addition, higher ratios led to instability during storage, resulting in visible precipitation within two weeks (**Supplementary Figure S2**). Therefore, subsequent formulations were prepared by dissolving 10 mg of DCPA and 1 mg of curcumin in 1 mL THF, which was then injected into deionized water to form Cur/DCPA-H_2_O liposomes. The curcumin loading content was quantified using UV-Vis spectroscopy (**Supplementary Figure S3A**), based on a calibration curve constructed from standard solutions (**Supplementary Figures S3B**, **C**), yielding a value of 3.25% ± 0.11%.

The average hydrodynamic diameters of DCPA-H_2_O and Cur/DCPA-H_2_O liposomes ranged from 85 to 110 nm, with a PDI of approximately 0.24 (**Figure 1B**). Both liposomes exhibited uniform spherical morphology by TEM (**Figure 1C**). Zeta potentials of both liposomes shifted from negative to positive when the pH decreased below 7.0 (**Figure 1D**), consistent with pH-responsive charge reversal. A negative zeta potential at physiological pH is critical for maintaining colloidal stability under normal biological conditions (Allam et al., 2013), whereas the charge reversal to a positive zeta potential under acidic conditions enables can facilitate association with cariogenic biofilm infection sites (Wang et al., 2022a). This indicates that the incorporation of curcumin into the bilayer of DCPA-H_2_O liposomes does not affect their surface physicochemical properties. Also, the DCPA-H_2_O liposomal matrix does not compromise spectral absorption properties (see **Figure 1E**) and the photodynamic performance of curcumin. Under light irradiation, the encapsulated curcumin efficiently generates ROS at a rate comparable to that of free curcumin (see **Figures 1F, G**), and even exhibits higher overall ROS yield (**Figure 1H**). All experiments were performed in triplicate with independently prepared liposome batches.

### 3.3 Antibacterial activity of the Cur/DCPA-H_2_O liposomes against planktonic dental caries cariogenic bacteria

**Figure 2:**
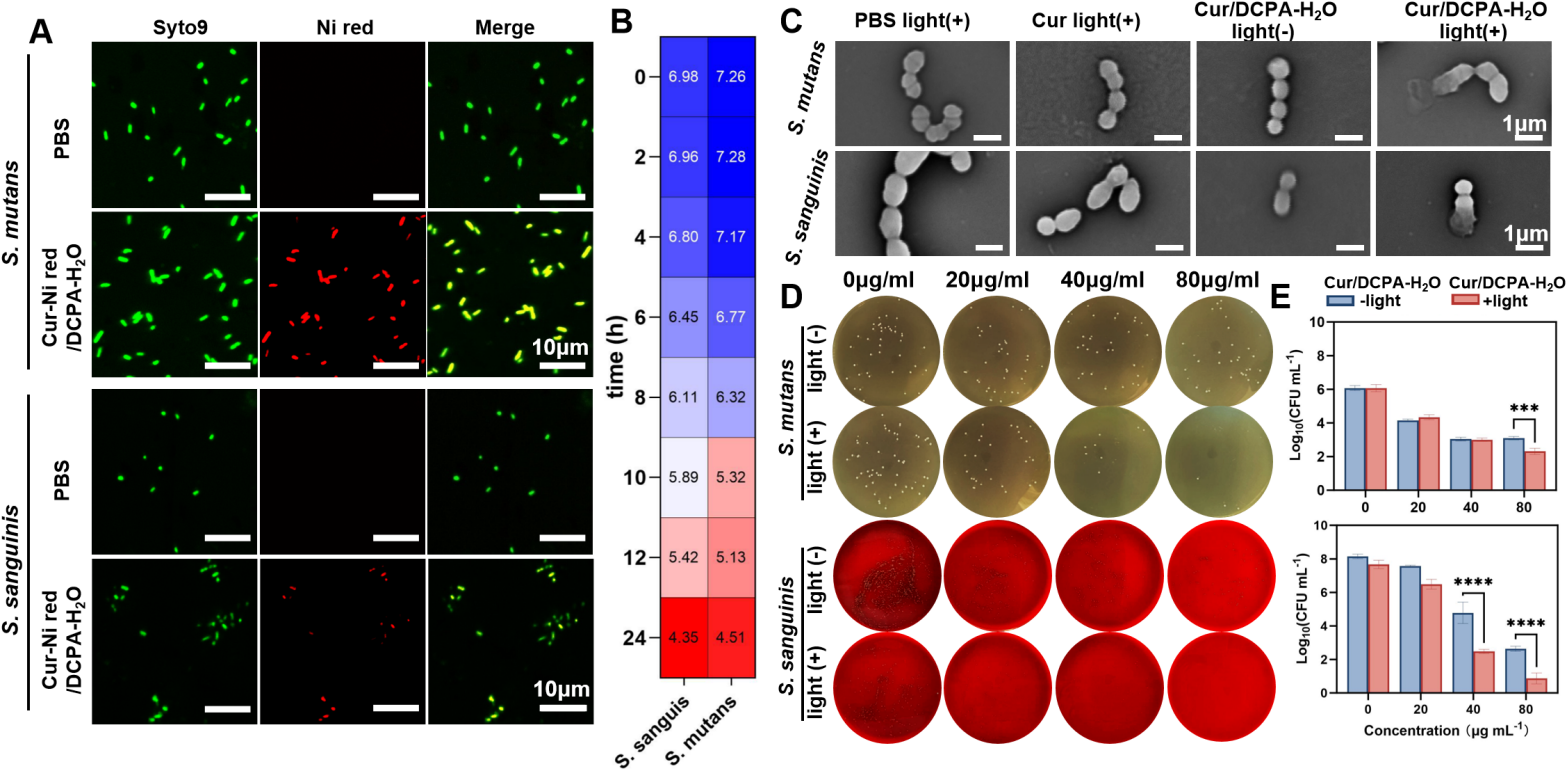
Antibacterial activity of the Cur/DCPA-H_2_O liposomes against planktonic dental caries cariogenic bacteria. **(A)** Nile red loaded-Cur/DCPA-H_2_O liposomes pH-dependent electrostatic interactions between Nile red loaded-Cur/DCPA-H_2_O liposomes and *S. mutans UA159* and *S. sanguinis BNCC354356*. Fluorescence micrographs of *S. mutans* and *S. sanguinis* and Nile red-loaded Cur/DCPA-H_2_O liposomes after coculturing for 2 h, together with a merged-channel overlayer image, showing interacting liposomes and *S. mutans* and *S. sanguinis* as yellow pixels. **(B)** pH value of dental caries cariogenic bacterial culture medium as a function of culture time. **(C)** SEM images of *S. mutans* and *S. sanguinis* after exposure to PBS, DCPA-H_2_O liposomes suspended in PBS, suspended in PBS Cur/DCPA-H_2_O liposomes. After exposure, bacteria were irradiated for 15 min with visible light (28 mW cm^-2^). **(D)** the agar plate of *S. mutans* and *S. sanguinis* after exposure to PBS and a series of Cur/DCPA-H_2_O liposomes suspended in PBS. **(E)** Calculated number of bacterial CFUs per mL for described in panel D. Data represent averages over triplicate measurements with separately prepared batches of nano-generators and differently grown biofilms and error bars denoting standard deviations. Asterisks indicate statistical significance (Student t-test) at ***p < 0.01, and ****p < 0.0001.

The antibacterial activity of Cur/DCPA-H_2_O liposomes *in vitro* was subsequently evaluated *S. mutans* and *S. sanguinis*, the principal cariogenic bacteria implicated in dental caries, were selected as representative pathogens. Coculturing planktonic *S. mutans* and *S. sanguinis* with Cur/DCPA-H_2_O liposomes, respectively, the experiments revealed that the liposomes could target and adhere to the bacterial surfaces (**Figure 2A**). This interaction is attributed to the metabolic production of lactic acid by *S. mutans* and *S. sanguinis*, which lowers the pH of the culture medium during incubation (**Figure 2B**). The resulting acidic environment can induce protonation of the liposomes, conferring a positive charged surface that facilitates electrostatic binding to the negatively charged bacterial membranes (Scheeder et al., 2023), thereby promoting the association of liposomes with bacterial surfaces. Following coculturing, the samples were irradiated with visible light (28 mW cm^-2^, 15 min). Upon irradiation, the morphology of both bacterial strains exhibited pronounced structural alterations, whereas no morphological changes occurred in the absence of light exposure (see **Figure 2C**). These findings suggest that the antibacterial effect is likely associated with ROS generation from curcumin encapsulated within the Cur/DCPA-H_2_O liposomes. This ROS-related antibacterial activity is further supported by a significant reduction in colony-forming units (CFUs) (**Figures 2D, E**). Moreover, the antibacterial efficacy of Cur/DCPA-H_2_O liposomes displayed a concentration-dependent behavior, with markedly greater inhibition observed at 80 μg mL^-1^ compared to 40 μg mL^-1^ or 20 μg mL^-1^. In contrast, negligible antibacterial activity was detected in the absence of light irradiation, confirming that Cur/DCPA-H_2_O liposomes without visible light irradiation, like curcumin alone even in the presence of visible light irradiation, were unable to cause significant damage to the bacterial integrity (also see **Figures 2C–E**). This can be attributed to its low solubility in aqueous solution and lack of targeting ability to bacteria. Data represent means ± SD from at least three independent experiments.

### 3.4 Anti-biofilm activity of Cur/DCPA-H_2_O liposomes against dental caries cariogenic bacteria biofilm *in vitro*

**Figure 3:**
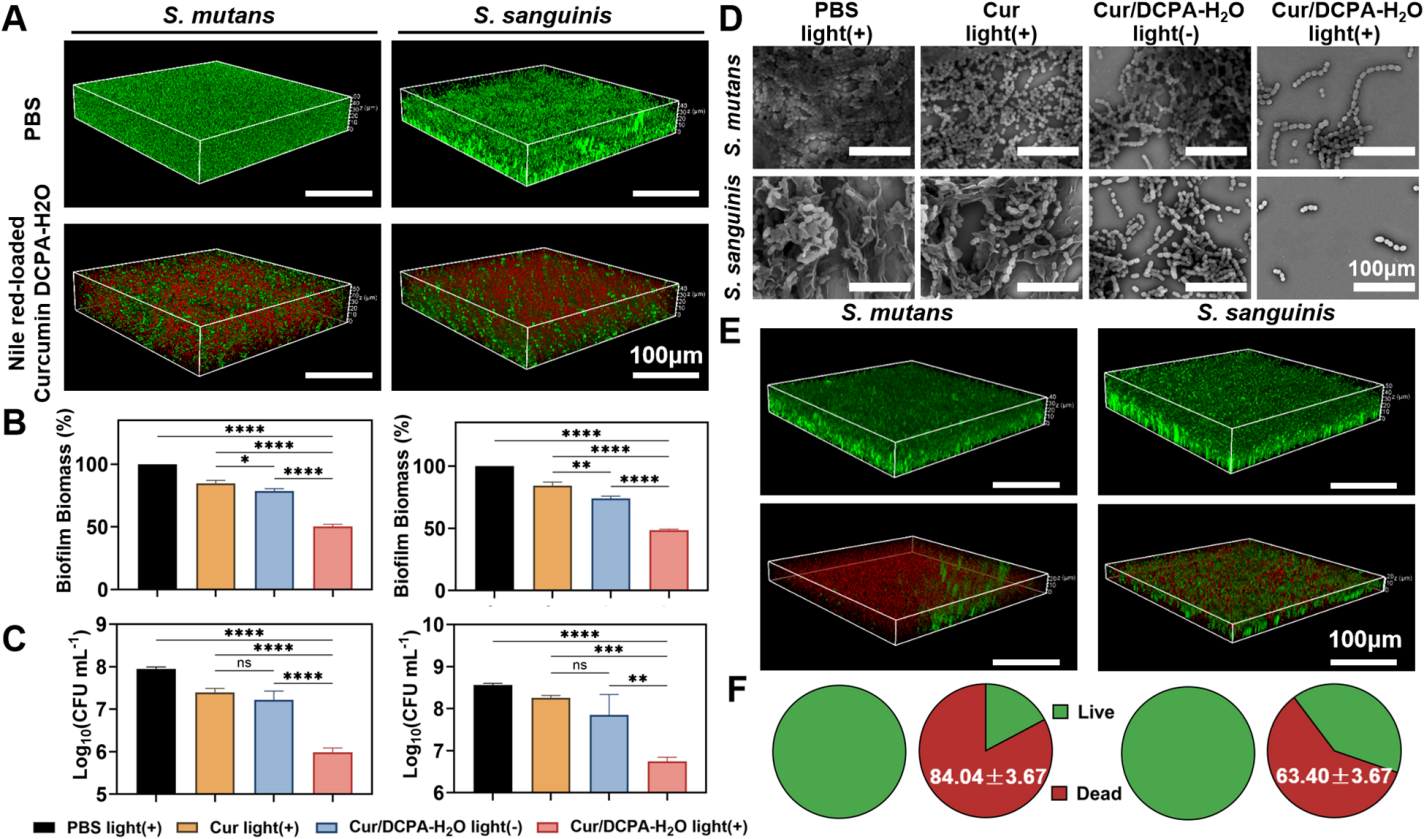
Anti-biofilm effects on 48-h *S. mutans UA159* and *S. sanguinis BNCC354356* biofilms by Cur/DCPA-H_2_O liposomes *in vitro*. **(A)** CLSM images of 48-h *S. mutans* and *S. sanguinis* biofilms exposed to Nile red-loaded Curcumin DCPA-H_2_O (5 μM Nile red) for 2 h. After exposure, the biofilm was washed twice with PBS, followed by staining with green-fluorescent SYTO 9 for 15 min in the dark and washed twice using PBS before CLSM imaging. **(B)** Biofilm mass of biofilms as described in panel C (left: *S. mutans*; right: *S. sanguinis*), CV staining, referred to **Supplementary Figure S4A**. **(C)** Same as panel B, but now for CFUs per mL, referred to **Supplementary Figure S4B**. **(D)** As same as panel C, now for SEM micrographs of *S. mutans* and *S. sanguinis* biofilm left after exposure. **(E)** As same as panel A, now for exposure to Cur/DCPA-H_2_O liposomes for 12 h, biofilms were irradiated for 15 min with visible light (28 mW cm^-2^), followed by staining with green-fluorescent SYTO 9 and red fluorescent PI for 15 min in the dark and washed twice using PBS before CLSM imaging. **(F)** The relative intensity of red and green fluorescence of *S. mutans* and *S. sanguinis* biofilms derived from CLSM images as in panel E. All error bars denote standard deviations over triplicate measurements with separately prepared liposome suspensions and bacteria. Asterisks indicate statistical significance at *p < 0.05, **p < 0.01, ***p < 0.001, ****p < 0.0001 and ns, no significance.

After evaluation the antibacterial efficacy of Cur/DCPA-H_2_O liposome against planktonic *S. mutans* and *S. sanguinis*, its anti-biofilm effects were further evaluated *in vitro*. 48-h biofilms, with thicknesses of 51 μm and 45 μm for *S. mutans* and *S. sanguinis*, respectively, were exposed to Cur/DCPA-H_2_O liposomes for 2 h. Due to the excellent self-targeting ability toward acidic bacterial biofilms (Wang et al., 2022b), the liposomes penetrated the entire biofilm (**Figure 3A**). In the Cur/DCPA-H_2_O group without irradiation, modest reductions in biomass and CFU counts were observed (approximately 20% biomass reduction and 1-log CFU reduction). Upon visible-light irradiation (28 mW cm^-2^, 15 min), greater reductions were observed, with biomass decreasing by nearly 50% and CFU counts by approximately 3 logs (see **Figures 3B, C**). Interestingly, free curcumin alone exposure to biofilm resulted in negligible effects, even under light irradiation (also see **Figures 3B, C**; **Supplementary Figure S4**). Accordingly, the SEM images demonstrated that the biofilm integrity was markedly disrupted, leaving only minimal residual bacteria after exposure to Cur/DCPA-H_2_O liposomes followed by visible light irradiation. In contrast, treatment with curcumin alone produced almost no change in biofilm integrity, showing results comparable to the PBS control (**Figure 3D**). The Cur/DCPA-H_2_O group without light irradiation also demonstrated certain antibacterial efficacy. This is likely attributed to the quaternary ammonium functionality of the protonated Cur/DCPA-H2O liposomes, which enables biofilm dispersal (Wang et al., 2022b), which facilitates biofilm dispersion, thereby enabling curcumin to exert antibacterial effects. Collectively, these results suggest that Cur/DCPA-H_2_O liposomes effectively generate ROS within the biofilm upon visible light irradiation. This ROS generation is likely to disperse the biofilm matrix, and exert bactericidal activity against the major cariogenic pathogens *S. mutans* and caries-associated pathogenic bacteria *S. sanguinis*. Notably, as *S. sanguinis* is generally considered a commensal species, the ecological impact of this treatment requires further investigation.

To further evaluate the potential role of ROS in the antibiofilm activity of Cur/DCPA-H_2_O under light irradiation, a Live/Dead assay was performed. Following treatment, biofilms were stained with SYTO 9 (green) and propidium iodide (PI, red) to visualize live and membrane-compromised bacteria, as well as overall biofilm structure and thickness. CLSM images (**Figure 3E**) showed that exposure to Cur/DCPA-H_2_O liposomes followed by visible light irradiation sharply reduced the biofilm thickness of both *S. mutans* and *S. sanguinis* from approximately 45 µm to 20 µm. In addition, quantitative fluorescence analysis (**Figure 3F**) revealed that 84% of the remaining *S. mutans* cells and 63% of the remaining *S. sanguinis* cells within the residual biofilms were dead. These results suggest that the antibacterial effect of Cur/DCPA-H_2_O liposomes upon light irradiation may be associated with ROS generation, which can disrupt bacterial cell membranes and contribute to cell death. Future studies will employ more clinically relevant multispecies oral biofilms to further validate the therapeutic efficacy, and using ROS scavengers to provide conclusive mechanistic evidence of curcumin’s photodynamic antibacterial activity. All quantitative data are from at least three independent experiments with separately prepared liposomes and biofilms.

### 3.5 *Ex vivo* killing of oral biofilm collected from dental caries patients

**Figure 4:**
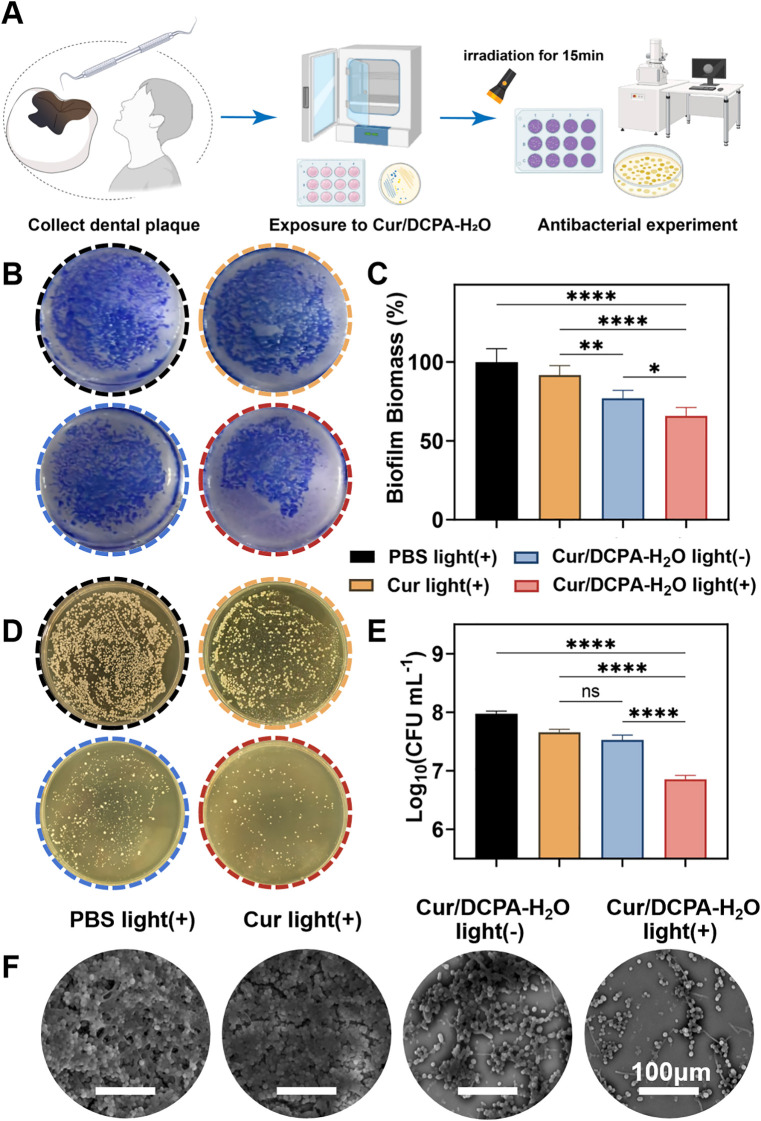
*Ex vivo* killing of bacteria of oral biofilms collected from dental caries patients. **(A)** Schematic illustration of the evaluation of the killing efficacy of Cur/DCPA-H_2_O liposomes against human dental caries biofilms *ex vivo*. **(B)** Photographs of caries samples exposed to different groups for 12 h, followed with or without visible-light irradiation (28 mW cm^-2^) for 15 min, then stained with CV for 30 min and washed with PBS. **(C)** Biofilm mass of biofilms as described in panel B, derived from CV staining, referred to panel B. **(D)** As same as panel B, but now for bacterial colonies on the agar plate. **(E)** Calculated number of bacterial CFUs per mL for described in panel D. **(F)** As same as panel B, but now for SEM micrographs of biofilms remaining after exposure. All error bars represent standard deviations from triplicate measurements using independently prepared liposome suspensions and biofilms. Asterisks indicate statistical significance at *p < 0.05, **p < 0.01, ****p < 0.0001 and ns, no significance.

Considering the physiological relevance *in vivo* and the controllability of experiments *in vitro* and biofilms extracted from dental caries are mainly composed of *S. mutans* (Han et al., 2025) can better simulate the actual oral conditions (Jiang et al., 2024). Hence, after the elimination of the cariogenic bacteria biofilm *in vitro*, the killing efficacy of the oral biofilm collected from dental caries patients was evaluated. After approval of the study by the Ethics Committee of the Tianjin Stomatological Hospital (PH-2023-J-025). Dental caries plaques were collected from the caries lesion of dental caries patients at the Department of Operative Dentistry and Endodontics in Tianjin Stomatological Hospital. Following, the plaques were exposed to the suspension of Cur/DCPA-H_2_O, curcumin solution and PBS mimicking the saliva, respectively (**Figure 4A**). After exposure to Cur/DCPA-H_2_O liposomes and visible-light irradiation (28 mW cm^-2^, 15 min), as same as the *in vitro* results, the biofilm biomass and CFU counts decreased relative to PBS group (see **Figures 4B–E**). Interestingly, the magnitude of reduction was lower than that observed in single-species *in vitro* models, consistent with the greater ecological complexity of clinical plaque. This ability was further confirmed by observing residual micrographs of plaque biofilm exposure to Cur/DCPA-H_2_O. As shown in **Figure 4F**, although the biofilm matrix was disrupted, the number of residual bacteria was relatively higher than that observed in **Figure 3D**. Cur/DCPA-H_2_O not only exhibited bactericidal activity but also maintained the homeostasis of microbial abundance and diversity (**Supplementary Figure S5A**). It significantly reshaped the oral microbial community structure (**Supplementary Figure S5B**) and reduced the abundance of caries-associated bacteria, including Klebsiella quasipneumoniae, Prevotella buccae, and unclassified Enterobacteriaceae, while preserving beneficial symbiotic bacteria (**Supplementary Figure S5C**). Considering the dynamic changes in clinical cariogenic bacterial samples during culture, these results require further investigation. Importantly, preserving part of the native microbiota may help maintain oral microbial balance, which is essential for oral health. Taken together, this strategy offers a promising approach for controlling the progression of clinical dental caries while avoiding complete eradication of the oral microbiome.

### 3.6 Evaluation of the therapeutic efficacy of Cur/DCPA-H_2_O liposomes on dental caries *in vivo*

Encouraged by the ability of Cur/DCPA-H_2_O liposomes to eliminate cariogenic bacterial biofilms in both *in vitro* and *ex vivo* experiments, we next evaluated their therapeutic efficacy against dental caries *in vivo*. Prior to this study, the biosafety of Cur/DCPA-H_2_O liposome was explored *in vitro*. As shown in **Supplementary Figures S6A, B**, the viability of the mouse fibroblast cell line L929 cells remained above 80% when co-cultured with Cur/DCPA-H_2_O liposomes at concentrations below 80 μg mL^-1^ under dark conditions. Consistently, liposomes at 80 μg mL^-1^ did not adversely affect cell integrity or morphology (see **Supplementary Figure S6D**). In addition, the hemolysis rate remained below 5% (**Supplementary Figure S6C**). These results indicate that Cur/DCPA-H_2_O liposomes at 80 μg mL^-1^ exhibit low cytotoxicity. Based on the above result, the concentration of 80 μg mL^-1^ was used for evaluating the therapeutic efficacy of Cur/DCPA-H_2_O liposomes on dental caries *in vivo*.

**Figure 5:**
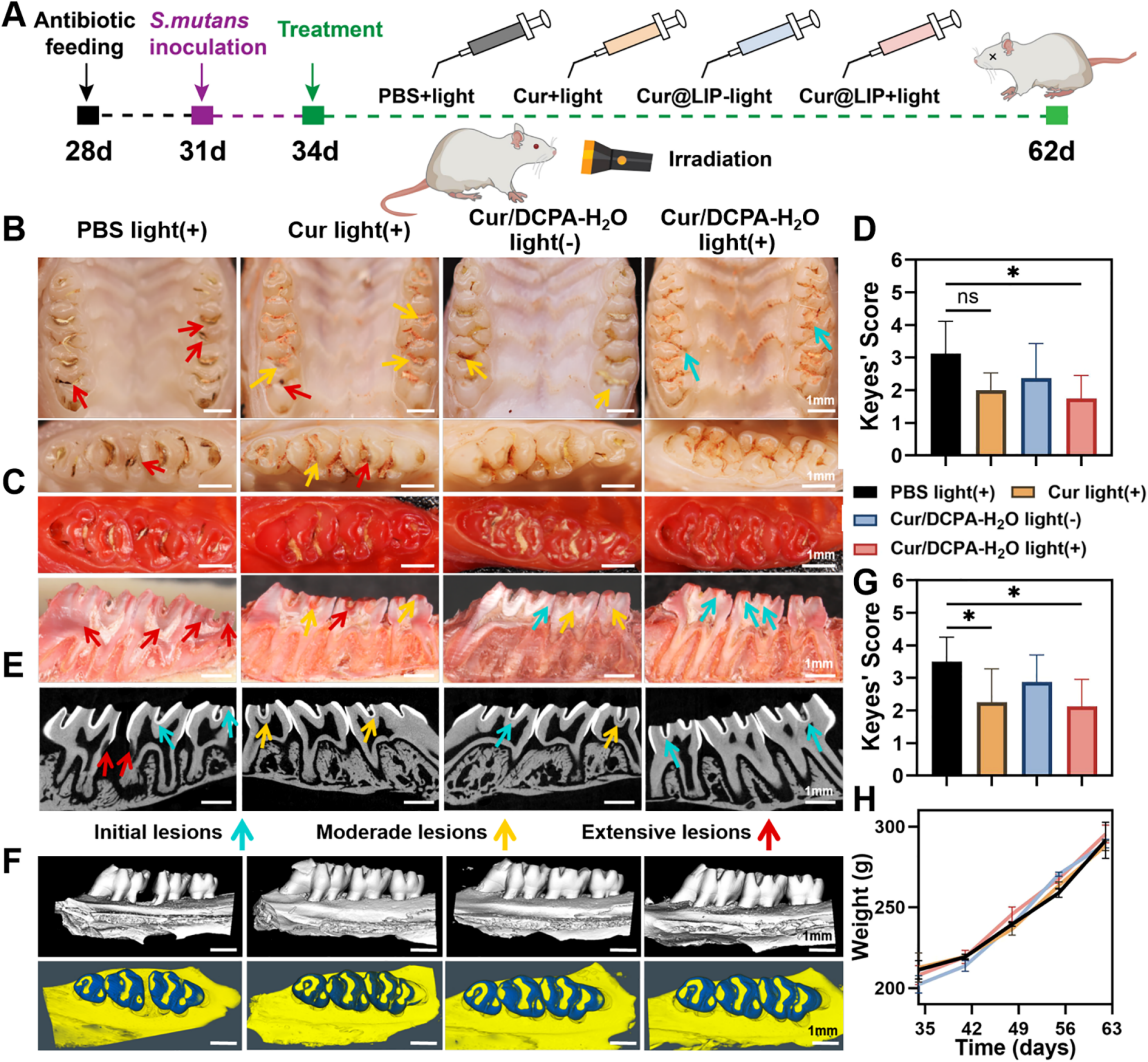
Therapeutic efficacy of Cur/DCPA-H_2_O liposomes on dental caries *in vivo*. **(A)** Schematic illustration of the dental caries rats and the treatment protocol using Cur/DCPA-H_2_O liposomes. **(B)** Representative stereoscopic microscopy images of the occlusal surfaces of rat teeth. **(C)** Representative images of carious lesions on smooth and sulcal surfaces, stained with murexide, showing different severities. **(D)** Quantitative analysis of Keyes' scoring for murexide-stained tooth surfaces, based on lesion depth and extent (1 - enamel only. 2 - slightly dentinal (<1/4 of the dentin region). 3-moderate dentinal (1/4 - 3/4 of the dentin region). 4-extensive dentinal (>3/4 of the dentin region)). **(E)** 2D sagittal micro-CT images of maxillary molars with carious lesions indicated by red arrows. **(F)** 3D reconstructed micro-CT images of maxillary molars from different treatment groups, with enamel highlighted in blue (density threshold >11,000 Hounsfield units). **(G)** Quantitative analysis of Keyes' scoring for 2D sagittal micro-CT images of molars, based on lesion depth and extent (1 - enamel only. 2 - slightly dentinal (<1/4 of the dentin region). 3 - moderate dentinal (1/4-3/4 of the dentin region). 4 - extensive dentinal (>3/4 of the dentin region)). **(H)** Body weight of rats monitored throughout the experiment. All data are presented as means ± standard deviations for 5 rats per group. Asterisks indicate statistical significance over comparisons indicated by the spanning bars at *p < 0.05; ns, no significance.

The therapeutic efficacy of topically applied Cur/DCPA-H_2_O liposomes was evaluated using a validated rodent caries model that closely replicates key oral conditions, including dietary exposure, salivary influences, host cellular responses, and hydrodynamic forces within the oral cavity (Li et al., 2025b). Cariogenic biofilms were established by inoculating rats with *S. mutans* in conjunction with a high-sucrose diet (see **Figure 5A**). As previously reported, the experimental procedure began with the administration of antibiotic-supplemented feed and water to eliminate endogenous *S. mutans* prior to inoculation (Lu et al., 2023). This antibiotic treatment phase was conducted for three consecutive days. After confirming the clearance of endogenous bacteria via oral swab sampling (**Supplementary Figure S7**), exogenous *S. mutans* inoculation was performed for the next three consecutive days, with administration twice daily. Once infection was successfully established, the protocol mimicked human oral hygiene habits by topically applying the solutions (100 μL orally) once daily starting on day 34, with a 1-minute coating time to simulate mouthwash use. After one month of treatment, the rats were sacrificed, and the molars were examined under a stereomicroscope (see **Figure 5B**). To enhance visualization of the damaged areas, murexide staining was applied to delineate the extent and depth of dental caries lesions. As shown in **Figure 5C**, lesions were classified into three grades: Initial lesions, affecting slight dentin, within 1/4 of the dentin; Moderate lesions, affecting moderate dentin, involving 1/4-3/4 of the dentin; and Extensive lesions, affecting extensive dentin, beyond 3/4 of the dentin. In the group of irrigation with PBS, even upon light irradiation, the most severe smooth-surface and fissure-cavity caries were observed. However, the group of irrigation with Cur/DCPA-H_2_O liposomes without light irradiation exhibited moderate reductions in caries severity, whereas the group of irrigation with Cur/DCPA-H_2_O liposomes upon light irradiation showed a significant decrease in both the incidence and severity of caries, consistent with the results of Keyes’ scoring (**Figure 5D**).

Moreover, dental caries is characterized by the destruction of dental hard tissue. Cariogenic bacteria in dental plaque metabolize dietary carbohydrates, producing acids that demineralize tooth surfaces (Chen et al., 2024). Consequently, carious lesions were evaluated using Keyes’ scoring and micro-CT analysis. In the group of irrigation with PBS with light irradiation, exhibited numerous enamel demineralization sites, some extending near the pulp, indicating severe caries. In contrast, the group of irrigation of Cur/DCPA-H_2_O liposomes upon light irradiation, no significant demineralization sites were observed (see **Figure 5E**), consistent with Keyes’ scoring results (**Figure 5G**). This further confirms the potent anti-cariogenic effect of Cur/DCPA-H_2_O with light irradiation. Importantly, teeth in the group of irrigation with Cur/DCPA-H_2_O liposomes upon light irradiation retained more intact enamel (blue), which was carefully stripped and reconstructed from the maxillary molars (**Figure 5F**). These findings underscore the material’s potential therapeutic effects in inhibiting caries progression and promoting remineralization. Additionally, no significant differences in body weight (**Figure 5H**) or organ histology (HE staining) were observed among the groups (**Supplementary Figure S8**), demonstrating that Cur/DCPA-H_2_O is safe *in vivo*.

### 3.7 Homeostasis of oral microbiome environment after the treatment of Cur/DCPA-H_2_O

**Figure 6:**
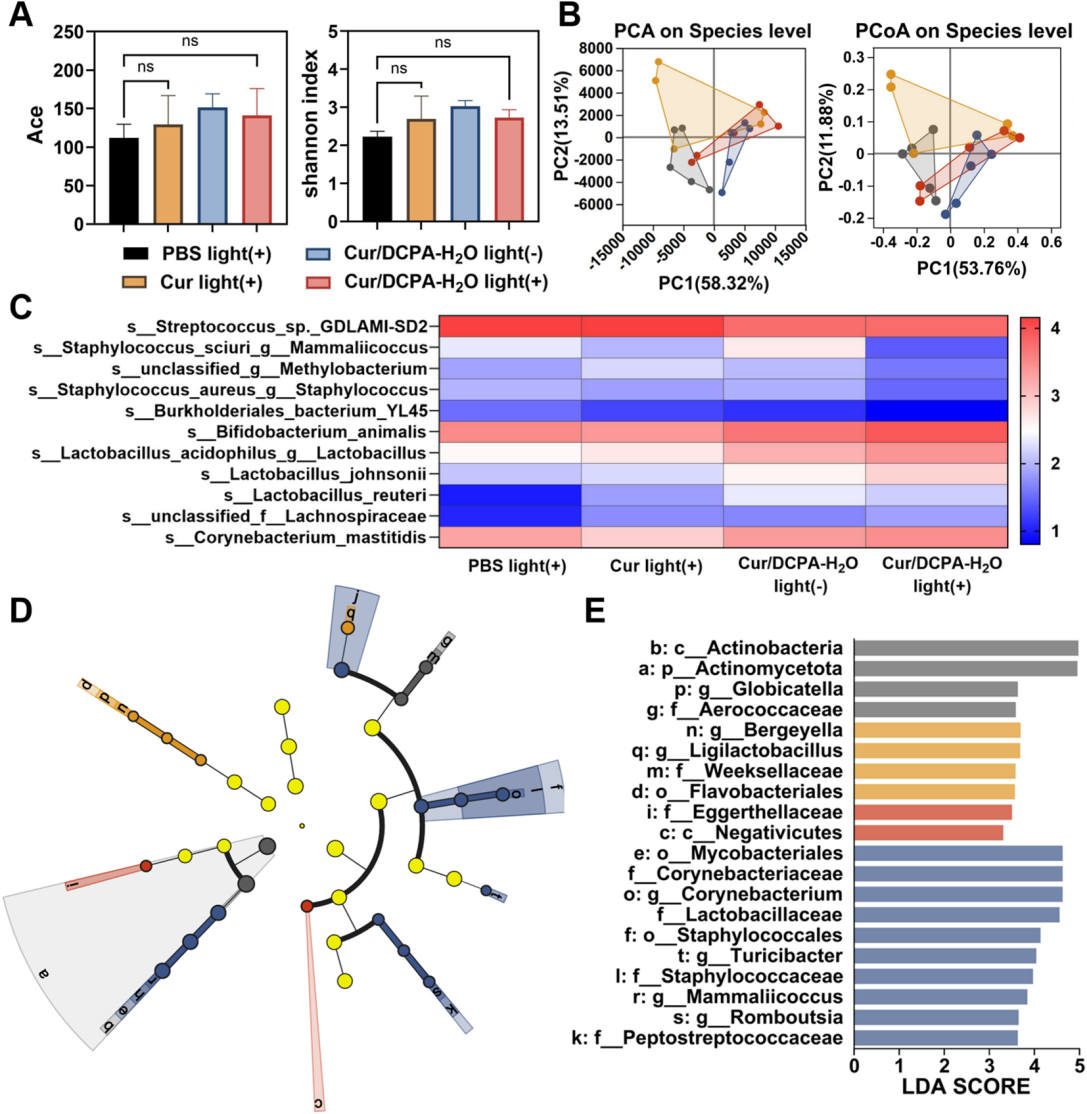
Impact of Cur/DCPA-H_2_O liposomes on oral microbiome homeostasis. **(A)** α-diversity of the oral microbiota at the species level, assessed by the Ace and Shannon indices. **(B)** β-diversity of the oral microbiota, illustrated by PCA and PCoA analyzes. **(C)** Heatmap showing the distribution of oral microbes at the species level. **(D)** LEfSe analysis identifying dominant bacteria under different treatments. Colored nodes indicate microbial groups significantly enriched in their respective clusters, while light yellow nodes represent groups with no significant differences across clusters. **(E)** Taxa ranked by LDA scores determined via LEfSe analysis. LDA (log10) > 2 and P < 0.05 indicate significantly higher relative abundance in the corresponding group compared to others. All data are presented as means ± standard deviations for 5 rats per group. Asterisks indicate statistical significance over comparisons indicated by the spanning bars at *p < 0.05; ns, no significance.

The ecological balance of the oral microbiota is a critical determinant of oral health (Baker et al., 2024). To assess whether Cur/DCPA-H_2_O liposomes disrupt this balance, 16S rRNA sequencing was employed to analyze the oral microbiota. This allowed comparison of the microbial composition and diversity in dental plaque between caries-afflicted rats and those treated with Cur/DCPA-H_2_O, thereby evaluating treatment effects in caries models. As shown in **Figure 6A**, no statistically significant differences in microbial diversity were observed among groups. The reduction in diversity in the caries rats may be attributed to the suppression of acid-sensitive bacteria (Shao et al., 2023). In Cur/DCPA-H_2_O, partially recovered microbial diversity relative to the caries model group. It suggests a shift in microbial composition under treatment conditions may partially mitigate dysbiosis. Principal component analysis (PCA) and principal coordinates analysis (PCoA) revealed distinct clustering of the Cur/DCPA-H_2_O light-treated group, clearly separated from the PBS light-irradiated group, whereas other treatment groups partially overlapped with the caries model group (see **Figure 6B**). Notably, Cur/DCPA-H_2_O upon light irradiation significantly reduced the abundance of cariogenic pathogens, including Streptococcus spp., Staphylococcus spp., Methylobacterium spp., as well as Burkholderiales_spp., which are associated with cavity formation. In contrast, beneficial oral bacteria, i.e., Bifidobacterium spp., Lactobacillus spp., Lachnospiraceae spp., and Corynebacterium spp. remained stable or even increased in abundance (see **Figure 6C**). This species-level rebalancing of the microbiota is consistent with the observed anti-caries effects. Supporting this, the cladogram (**Figure 6D**) and LDA score analysis (**Figure 6E**) revealed that treatment with Cur/DCPA-H_2_O liposomes under light irradiation enriched taxa associated with oral health, including members of the Eggerthellaceae family. Because a healthy baseline group was not included, these changes suggested that Cur/DCPA-H_2_O liposomes rebalance toward a less dysbiotic profile, reduced the relative abundance of several acidogenic taxa while preserving or increasing several commensal genera. This property is particularly noteworthy, as it differs from current antibiotic-based anti-caries materials that face significant challenges, i.e., bacterial resistance and disruption of the oral microbiota during prolonged use (Gao et al., 2024). Moreover, this liposomal curcumin delivery system mitigates the indiscriminate bactericidal effects observed in conventional curcumin-mediated photodynamic therapy (Schone et al., 2025). We attributed this to the ability of Cur/DCPA-H_2_O to preferentially target acidic dental caries biofilms, which are primarily composed of *S. mutans* and *S. sanguinis* and characterized by lactic acid production during metabolism. Nevertheless, further quantitative measurement of lactate levels and direct validation of pH-dependent targeting under biologically relevant conditions are required to elucidate the antibacterial mechanism.

## 4 Discussion

In this study, we developed a curcumin-loaded DCPA-H_2_O liposomal system (Cur/DCPA-H_2_O) designed to enhance targeted delivery within the acidic microenvironments associated with dental caries. In the acidic environment, these nanoliposomes acquire a positive surface charge through protonation, which may promote pH-responsive accumulation and enhance association with negatively charged biofilms, potentially facilitating deeper penetration. Upon light irradiation, curcumin generated abundant ROS to disrupt the EPS matrix and kill embedded bacteria. The formulation reduced cariogenic biofilms *in vitro*, showed activity against *ex vivo* clinical plaque, lowered caries severity and reduced enamel demineralization in a rat model. Moreover, the system displayed excellent cytocompatibility and minimal hemolytic activity, indicating favorable biosafety for oral applications. The pH-responsive Cur/DCPA-H_2_O system in this study leverages acid microenvironment-triggered targeted delivery and photodynamically induced antibacterial effects to effectively eliminate cariogenic biofilms, thereby reducing the microbial imbalance risks associated with antimicrobial peptides and the nonspecific damage caused by metal ion antimicrobial agents.

However, while Cur/DCPA-H_2_O shows potential for targeted biofilm elimination and caries inhibition, several avenues remain for future exploration. First, an empty DCPA-H_2_O liposome control was not systematically included in the present study. Although our previous work has demonstrated that empty liposomes do not exhibit intrinsic antibacterial activity, we acknowledge that the absence of this control limits the ability to fully distinguish carrier-related effects from curcumin-mediated activity. Notably, the modest antibacterial effects observed in the Cur/DCPA-H_2_O group without irradiation suggest that charge-mediated interactions between liposomes and bacterial membranes may contribute to the overall antibacterial outcome. Therefore, the relative contributions of the carrier and the photodynamic activity of curcumin cannot be completely separated in this study and should be further clarified in future work. Second, the *in vitro* models used single-species *S. mutans* and *S. sanguinis* biofilms, although these are widely used for initial screening, they do not fully recapitulate the complexity of multispecies clinical plaque. It should also be noted that resuspension and incubation clinical *ex vivo* bacteria may alter the native microbial composition, limiting ecological interpretation. Future studies will employ clinically relevant multispecies oral biofilm models and optimized *ex vivo* protocols to address these limitations. Third, the 15-minute daily light irradiation used *in vitro*, while necessary to ensure sufficient ROS generation for photodynamic activity, may require optimization for clinical feasibility. Although ROS generation was confirmed using ABDA assays, direct evidence linking ROS to bacterial killing remains limited. Future studies incorporating direct lactate quantification and ROS scavengers experiments will be needed to establish causality. Fourth, long-term *in vivo* studies are essential to evaluate the durability of Cur/DCPA-H_2_O, its effects on oral microbial ecology, and the potential for repeated administration. Fifth, the system could be further engineered for multifunctionality, such as integration with additional remineralization agents, to improve therapeutic outcomes in advanced carious lesions. Finally, extending this platform to other oral biofilm-associated diseases, such as periodontitis or peri-implantitis, may broaden its clinical relevance. Collectively, these efforts will help advance Cur/DCPA-H_2_O as a next-generation, patient-friendly strategy for the management of dental caries and beyond.

## 5 Conclusion

Cur/DCPA-H_2_O demonstrated effective biofilm reduction and caries inhibition *in vitro*, *ex vivo*, and *in vivo* while showing good biocompatibility. It was established as a promising, biocompatible, and effective therapeutic strategy for caries prevention and management, offering a light-controlled, pH-responsive platform for future translational development and may contribute to next-generation, microbiome-friendly caries therapies.

## Data Availability

The original contributions presented in the study are included in the article/**Supplementary Material**. Further inquiries can be directed to the corresponding authors.
