# Supplementary material for: Biofilm-targeted liposomal curcumin delivery system for anti-caries therapy
Source: Front Cell Infect Microbiol. 2026 Apr 29;16:1808450. doi: 10.3389/fcimb.2026.1808450 (PMC13168170; doi:10.3389/fcimb.2026.1808450)
Supplement: Supplementary file 1 [file DataSheet1.pdf]

Supplementary Material

Biofilm-Targeted Liposomal Curcumin Delivery System for Anti-Caries Therapy

Yao Chen[1,4+], Jing Li[2,4+], Yongjian Sun[2,4+], Xin Yue[2,4], Xiao-Han, Tian[3], Feng, Liu[5], Da-Yuan Wang[3*] and Jing Shen[2,4*]

[1]Department of Operative Dentistry and Endodontics, Tianjin Stomatological Hospital, School of Medicine, Nankai University, Tianjin 300041, China

[2]Department of International VIP Dental Clinic, Tianjin Stomatological Hospital, School of Medicine, Nankai University, Tianjin 300041, China

[3]State Key Laboratory of Medicinal Chemical Biology, Key Laboratory of Functional Polymer Materials, Ministry of Education, Institute of Polymer Chemistry, College of Chemistry, Nankai University, Tianjin 300350, PR, China

[4]Tianjin Key Laboratory of Oral and Maxillofacial Function Reconstruction & Stomatology Institute of Nankai University, Tianjin 300041, China

[5]Key Laboratory of Industrial Fermentation Microbiology, Ministry of Education, College of Biotechnology, Tianjin University of Science and Technology, Tianjin 300457, P. R. China

+ Both first authors contributed equally.

*** Correspondence:**

Da-Yuan Wang: d.wang@nankai.edu.cn

Jing Shen: shenjing611@163.com

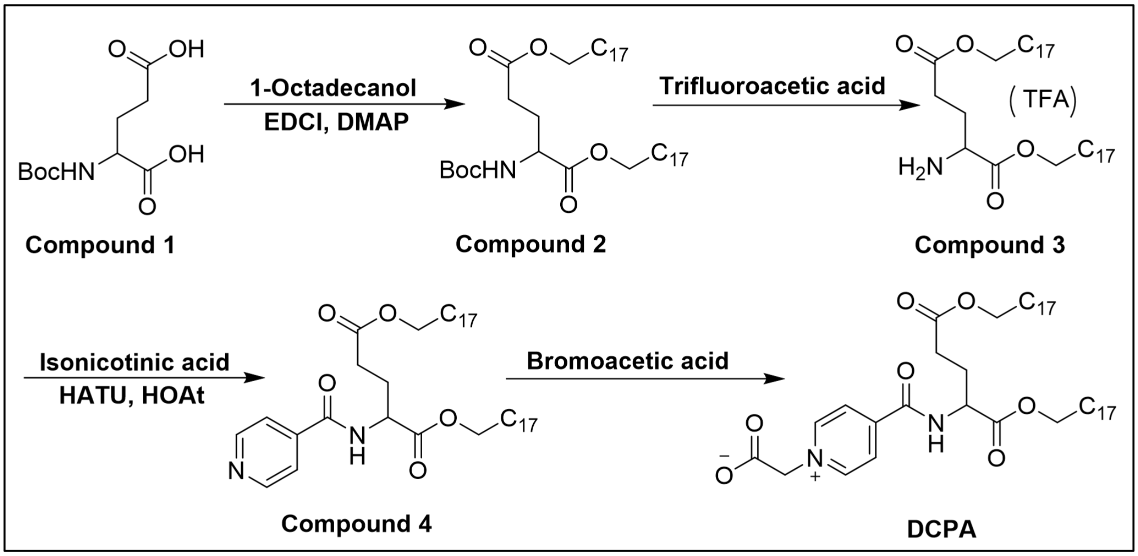

Scheme S1. Syntheses of 2-(4-((1,5-bis(octadecyloxy)-1,5-dioxopentan-2-yl) carbamoyl) pyridin-1-ium-1-yl) acetate (DCPA).

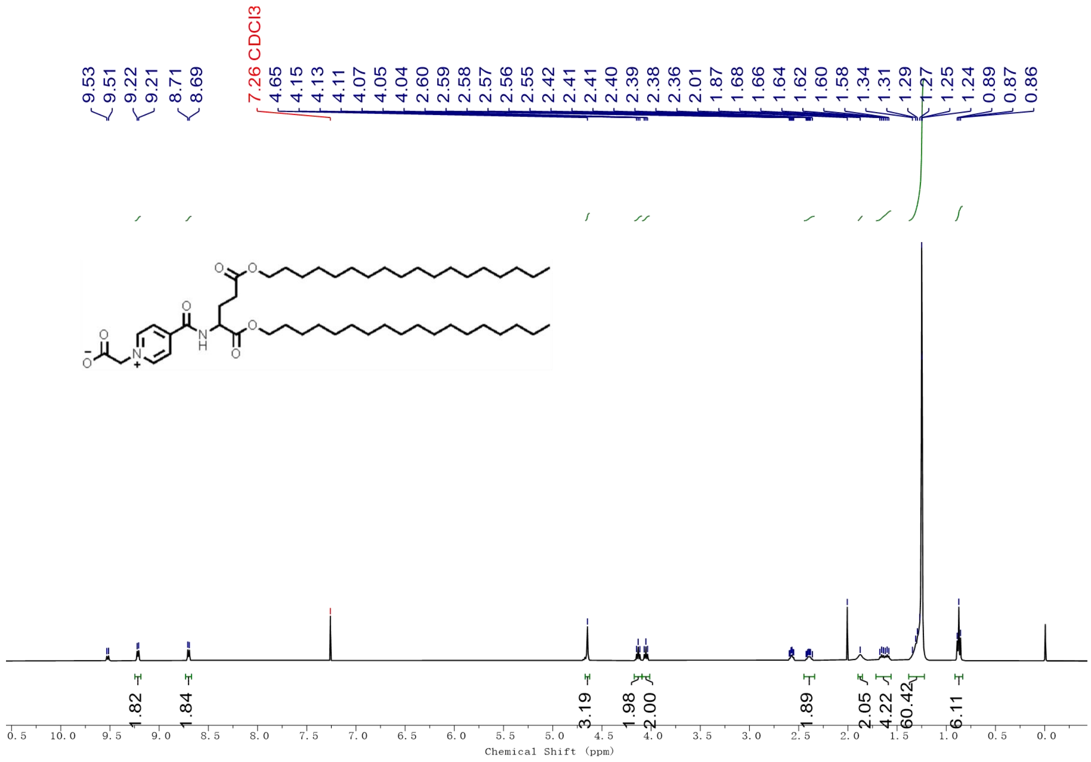

Figure S1. ^{1}H NMR spectrum of lipid DCPA in $CDCl_3$.

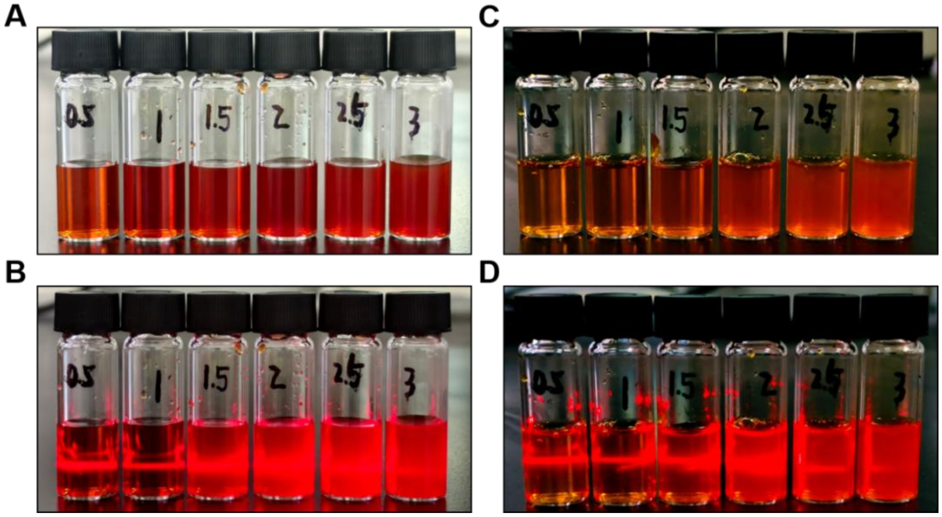

Figure S2. Comparison of the stability of DCPA-H_2O liposomes with different curcumin loading. **(A)** Images of Cur/DCPA-H_2O liposomes with different curcumin loading (wt%) after self-assembly on day 1. **(B)** As same as panel A, but now the Tyndall effect. **(C)** As same as panel A, but with liposomes stored in a dark environment for 14 days. **(D)** As same as panel B, but with liposomes stored in a dark environment for 14 days.

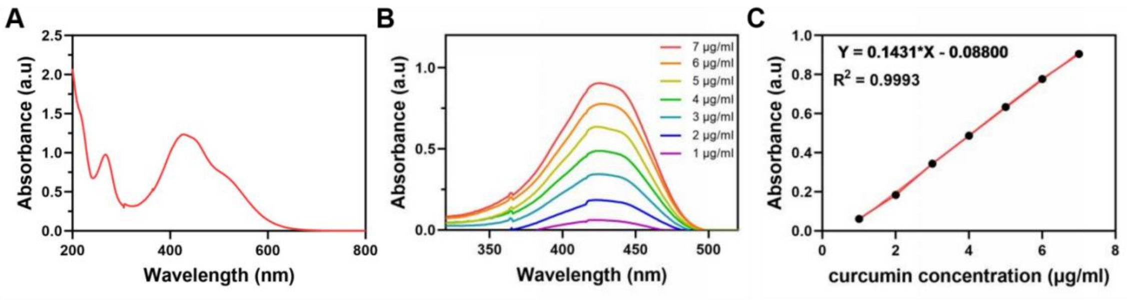

Figure S3. Absorbance as a function of the concentration of curcumin in DMSO. **(A)** Absorbance at 425 nm of curcumin. **(B)** UV-vis absorption spectra of curcumin in DMSO between 320-520 nm at different curcumin concentrations. **(C)** Calibration curve at the absorbance at 425 nm of curcumin as a function of the concentration, calculated from panel B.

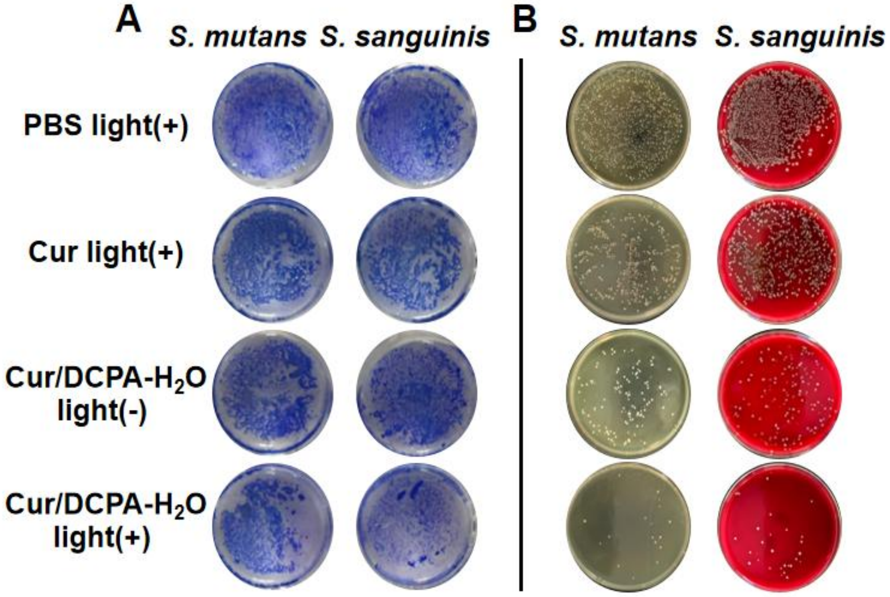

Figure S4. The anti-biofilm effect of Cur/DCPA-H_2O liposomes. **(A)** Images of the *S. mutans* and *S. sanguinis* biofilm after different treatment, followed by staining with Crystal violet staining of *S. mutans* and *S. sanguinis*. **(B)** Same as panel A, but now for images of bacteria growth on the agar plate.

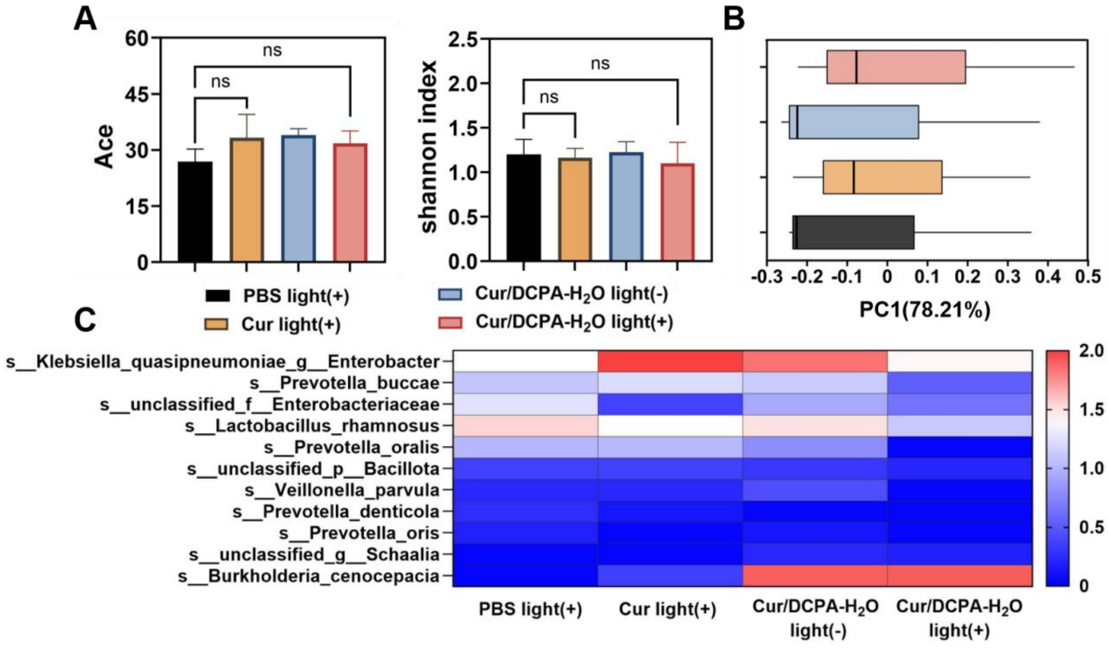

FigureS5. *Ex vivo* modulatory effects on the oral microbiome of Cur/DCPA-H_2O. **(A)** α-diversity of the oral microbiota, assessed by the Ace and Shannon indices. **(B)** β-diversity of the oral microbiota, illustrated by PCoA analysis. **(C)** Heatmap showing the distribution of oral microbes at the species level. All data are presented as means ± standard deviations for 5 samples per group. Asterisks indicate statistical significance over comparisons indicated by the spanning bars at $*p < 0.05$, ns, no significance.

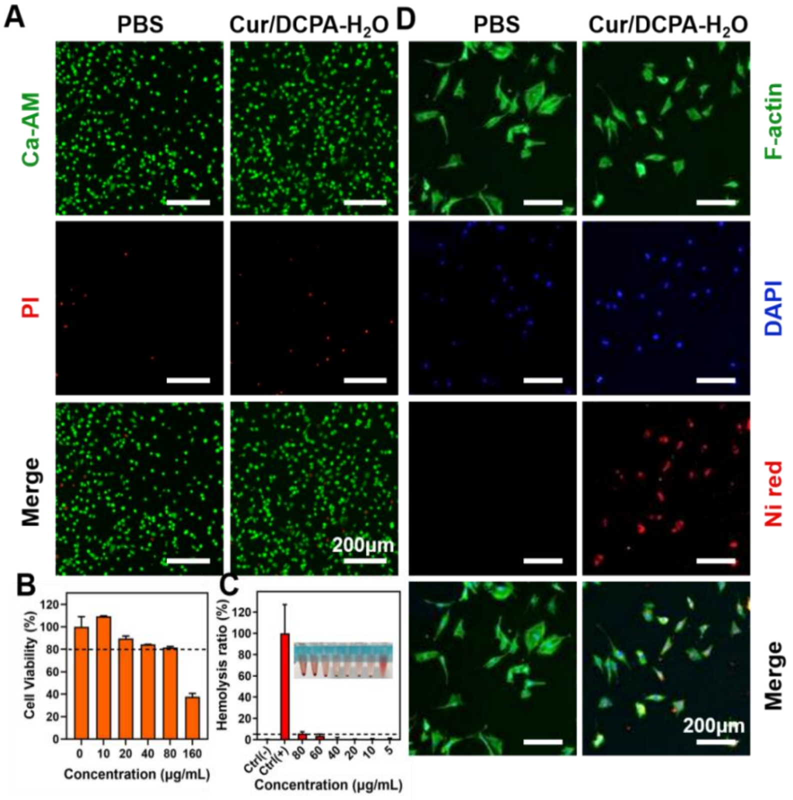

Figure S6. Biocompatibility of Cur/DCPA-H_2O *in vitro*. **(A)** Live/dead staining of L929 cocultured with PBS group and Cur/DCPA-H_2O (light+) group for 24 h. **(B)** Cell viability of L929 cocultured with Cur/DCPA-H_2O (light+) group for 24 h. **(C)** Hemolysis rate of Cur/DCPA-H_2O. **(D)** Phalloidin staining of MC-3T3-E1 with PBS group and Cur/DCPA-H_2O (light+) group for 24 h. All data are presented as means ± standard deviations for 3 samples per group. Asterisks indicate statistical significance over comparisons indicated by the spanning bars at *$p < 0.05$, **$p < 0.01$, ***$p < 0.001$, ****$p < 0.0001$; ns, no significance.

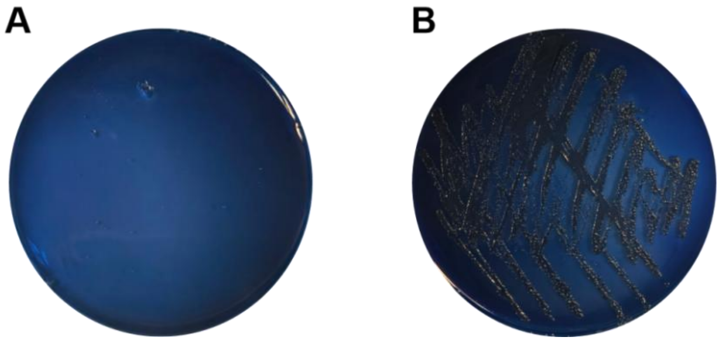

Figure S7. Determination of animal model establishment by plate streaking. Animals were screened for *S. mutans* infection by plating oral swabs on agar plates. **(A)** Oral swabs collected after antibiotic treatment. **(B)** Oral swabs collected after *S. mutans* inoculation to confirm successful infection.

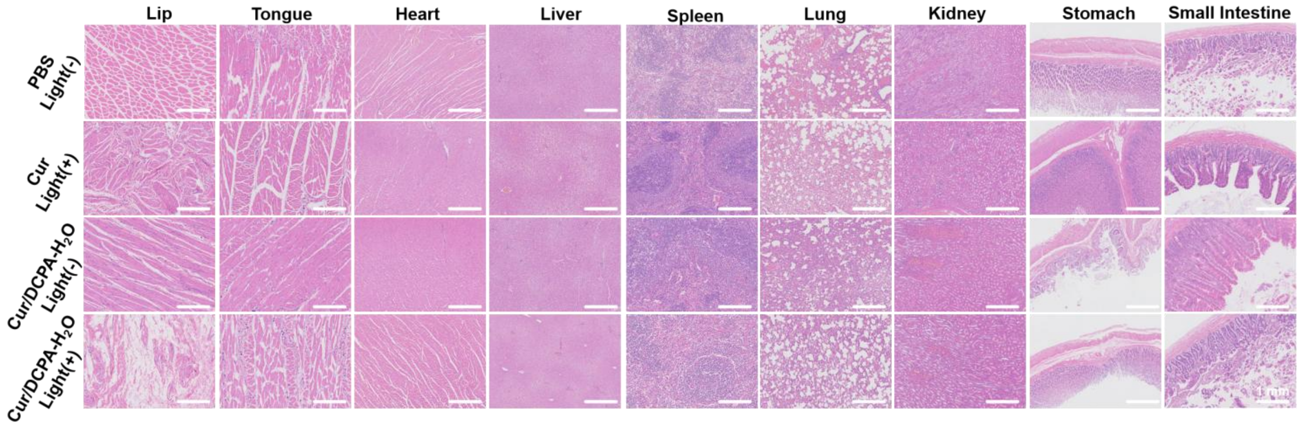

Figure S8. HE staining of nine organs in rats: lip, tongue, heart, liver, spleen, lung, kidney, stomach and small intestine.

Table S1. The hydrodynamic diameter and polydispersity index of DCPA-H_2O liposomes with different curcumin loading (wt%).

wt% curcumin	DCPA (mg)	Curcumin (mg)	Diameter (mm)		PDI	
			Day0	Day14	Day0	Day14
5%	10	0.5	98.93±0.96	113.83±0.60	0.20±0.01	0.21±0.01
10%	10	1.0	103.17±2.09	107.73±2.85	0.24±0.02	0.22±0.01
15%	10	1.5	124.77±0.66	123.67±0.12	0.36±0.01	0.35±0.02
20%	10	2.0	133.7±2.26	130.97±1.79	0.41±0.01	0.36±0.02
25%	10	2.5	148.73±3.04	147.6±5.91	0.46±0.01	0.40±0.03
30%	10	3.0	150.2±2.29	153.5±8.91	0.47±0.02	0.41±0.01
